# The heart knows best: baseline heart rate variability as guide to transcutaneous auricular vagus nerve stimulation in depression

**DOI:** 10.1038/s41398-025-03780-y

**Published:** 2025-12-06

**Authors:** Carmen Schiweck, Mareike Aichholzer, Emily Brandt, Moritz Schneider, Konrad Meyer, Tirage Hamzehloiya, Mishal Qubad, Carmen Uckermark, Leona Jacobsen, Kevin Amaral, Joyce Auer, Gianluca Bruno, Tong Zhao, Aicha Bouzouina, Susanne Schillo, Ruth Hanssen, Tim Hahn, Jonathan Repple, Silke Matura, Jonathan Kingslake, Andreas Reif, Sharmili Edwin Thanarajah

**Affiliations:** 1 Goethe University Frankfurt, University Hospital, Department of Psychiatry, Psychosomatic Medicine and Psychotherapy, Frankfurt, Germany; 2 Goethe University Frankfurt, Cooperative Brain Imaging Center - CoBIC, Frankfurt, Germany; 3 Ernst Strüngmann Institute for Neuroscience (ESI) in Cooperation with Max Planck Society, Frankfurt am Main, Germany; 4 University of Cologne Faculty of Medicine and University Hospital Cologne, Policlinic for Endocrinology, Diabetology and Preventive Medicine (PEPD), Köln, Germany; 5 Max Planck Institute for Metabolism Research, Cologne, Germany; 6 University of Münster, Institute for Translational Psychiatry, Münster, Germany; 7 P1vital Products Limited, Howbery Park, Wallingford, Oxfordshire UK; 8 Fraunhofer Institute for Translational Medicine and Pharmacology, Frankfurt, Germany

**Keywords:** Depression, Human behaviour, Predictive markers

## Abstract

Major Depressive Disorder can be conceptualized as a chronic stress condition associated with autonomic dysregulation, including blunted heart rate reactivity, elevated basal cortisol levels and higher low-grade peripheral inflammation, pointing to an autonomic imbalance with relative sympathetic overactivation and parasympathetic attenuation. Transcutaneous vagus nerve stimulation (taVNS) offers a non-invasive method to stimulate the vagus nerve, as key component of the parasympathetic system, to restore autonomic balance. Here, we examined whether emotional (i.e., positive and negative emotions), cardiac (i.e., heart rate and heart rate variability (HRV)), and inflammatory (i.e., TNF- α, IL-6) reactivity to stress are differentially influenced by taVNS in participants with MDD and controls. Additionally, we performed a post-hoc analysis with participants stratified by baseline RMSSD, as a proxy for vagus nerve activity, to evaluate the utility of biological stratification beyond diagnostic criteria. To assess the effect of chronic stress we conducted a single-blinded, cross-over, randomized controlled trial with 110 participants (51 controls and 59 MDD participants). For the analysis stratified by RMSSD group, we grouped participants into low (n = 54) vs. high (n = 55) RMSSD groups, regardless of diagnosis. All participants were subjected to an acute stress paradigm, both with taVNS and sham stimulation on two separate days, in a counter-balanced order. There was no difference in any of the outcomes regarding the effect of taVNS in participants with MDD and controls. Analyses split by RMSSD group, however, showed that for those with low RMSSD, taVNS restored the blunted cardiac stress response and numerically decreased TNF-α levels. Unexpectedly, in participants with high RMSSD, the opposite pattern was observed: heart rate and TNF-α were significantly increased, and vagally mediated heart rate variability was significantly decreased under taVNS compared to sham stimulation. Analyses using RMSSD as continuous predictor yielded similar results. Our findings suggest that RMSSD-based stratification may be a useful tool for predicting outcome of (ta)VNS treatment. We encourage researchers with HRV data to re-evaluate their findings through RMSSD stratification.

## Introduction

Major depressive disorder (MDD) is a highly prevalent and debilitating condition. Still, 30% of participants with MDD do not respond to treatment, highlighting the need for novel treatment options. In the search for new treatment modalities, the conceptualization of MDD as a condition of chronic stress is of interest [1–3]. Specifically, participants with MDD report excessive subjective levels of chronic stress and show corresponding physiological alterations, including autonomic dysregulation. This is evident in reduced heart rate variability (HRV) and impaired responsivity to acute stress on both emotional and physiological measures (e.g., cortisol resistance, higher heart rate, blunted autonomic reactivity) [4, 5]. Hence, specifically targeting biological pathways affected by chronic stress may offer new avenues for MDD treatment.

The vagus nerve (VN) - the principal parasympathetic component of the autonomous nervous system - is a key player in the stress response and recovery. Connecting the brain stem and the inner organs, the VN facilitates the bidirectional communication necessary for rapid adaptations after an acute stress exposure. VN efferents influence cardiac reactivity and inflammatory responses via multiple pathways including the cholinergic anti-inflammatory reflex [6–8]. Conversely, interoception and the emotional (and attentional) responses are integrated through strong projections that exist between the VN, brain stem nuclei (nucleus tractus solitarii and dorsal-motor nucleus), the hypothalamus, mesolimbic regions, and higher cortical areas including the insular and ventromedial prefrontal cortices [9–14]. Hence, impaired vagus function, indicated by low cardiac parasympathetic activity (CPA) [15] significantly affects the stress response and homeostasis. Currently - with the exception of the invasive ultrasound-guided microneurography - there is no method to assess vagal activity directly for humans. The current non-invasive standard to approximate CPA is through the root mean square of successive differences (RMSSD) or through Respiratory Sinus Arrythmia, derived from an electrocardiogram. RMSSD is a widely used time-domain measure of heart rate variability (HRV) that is thought to specifically index vagally mediated cardiac–parasympathetic activity during normal breathing patterns. Shaffer and Ginsberg [16] highlight that RMSSD is particularly well-suited for short-term recordings (~5 min or less) because it is relatively insensitive to respiratory influences (see also [15]) and reflects fast time-scale fluctuations in heart rate, making it an indicator of parasympathetic tone.

Given the VN’s key role in the stress response and recovery, it is noteworthy that (invasive) vagus nerve stimulation is an approved treatment for treatment-resistant cases of MDD. Indeed, VN stimulation (VNS) has shown promising results in reducing depressive symptoms, but the underlying mechanisms for this effect are not understood.

With the introduction of non-invasive techniques, evidence on the physiological mechanisms is accumulating. Unlike invasive VNS that activates both efferents and afferents in the cervical trunc, transcutaneous auricular VNS (taVNS) selectively stimulates sensory vagal afferents Iin the outer ear. This targeted approach has been shown to 1) elicit activation of vagal brain stem nuclei and the downstream projections in the brain [17–19], 2) influence learning, motivation and social behaviour [20–22], and 3) exert anti-inflammatory effects under specific conditions [23]. These latter effects are thought to be mediated predominantly through central mechanisms, as downstream effects in peripheral organs such as the heart or spleen result from brainstem activation rather than direct efferent stimulation.

taVNS has been demonstrated to influence emotional regulation during acute stimulation. Specifically, taVNS significantly enhanced positive emotions in healthy participants who exerted effort on low-reward tasks, and the effect was greater in participants with lower baseline levels of positive affect [24]. The impact of taVNS on peripheral inflammation remains inconclusive. While rodent studies have consistently demonstrated inflammation-attenuating effects of VNS [25], our recent meta-analysis of human studies [23] did not show a consistent effect, except for studies involving an acute inflammatory challenge. Methodological heterogeneity across studies and large diversity in participant samples complicates interpretation.

Finally, despite the crucial role of the autonomic system in cardiac stress reactivity, few studies (i.e., [26, 27]) have investigated the impact of taVNS on heart rate and heart-rate variability during acute stress, and to the best of our knowledge, no studies have investigated this in MDD. While generally positive effects of taVNS on depressive symptoms have been reported [28], studies on the mechanism of therapeutic action in MDD remain scarce.

Considering that (1) MDD is a chronic stress condition with reduced HRV [29], and reduced autonomic responses to acute stress [5] and (2) taVNS can selectively activate vagal afferents and modulate the central autonomic network, we hypothesized that taVNS would increase acute stress reactivity, restore blunted HRV, and reduce inflammatory response to acute stress in participants with MDD and healthy controls. In addition, given the previously reported effect on positive emotions, we aimed to explore if positive and negative emotions in response to a stress paradigm are influenced by taVNS. After analyzing the groups by diagnosis, we performed a stratification of participants by RMSSD, as a biological marker of cardiac vagal activity, and hypothesized that particularly participants with low RMSSD will benefit from taVNS compared to those with high RMSSD.

## Methods

### Ethical approval and pre-registration and trial design

The single-blind randomized controlled trial “MODULATE Depression” was approved by the Ethical Committee of the Goethe University Frankfurt (Approval number 2021-48) and pre-registered at the German Clinical Trials Register (Identifier: DRKS00024823) and can be retrieved in the WHO Clinical Trial Registration Platform.

**Fig. 1:**
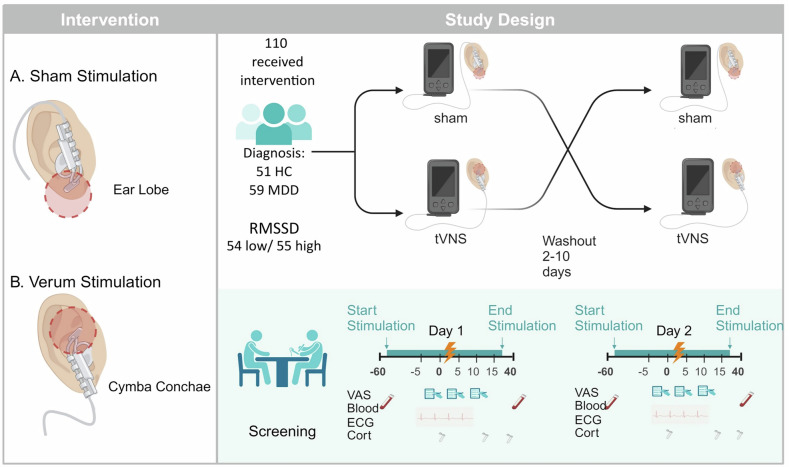
Study Design. Left panel: **A** Sham stimulation at the earlobe. **B** Verum stimulation at the right cymba conchae. Right panel: Illustration of cross-over, single-blind, randomized, sham-controlled trial. Stimulation was started after the blood sample (−60 min) and ended after the stress task. VAS scales were collected after the baseline (0 min), after stress exposure (5 min) and after recovery (5 min). A final blood sample was collected 40 min after stress exposure. Timescales are approximate and did vary according to time needed for completing experimental procedures such as VAS scales. Image created with Biorender.com.

All participants provided written informed consent followed by a screening visit, and two test-days. On the test days, sham stimulation or transcutaneous auricular vagus nerve stimulation (taVNS) were administered in a counter-balanced order (see Fig. 1.). On the screening day, participants were assessed for eligibility using the Mini-Neuropsychiatric International Interview (MINI 7.0.2 for DSM-5) and the Montgomery Asberg Depression Rating Scale (MADRS), completed various questionnaires (see below for details) and registered in the electronic patient-reported outcome system (P1vital® ePRO system). If eligible, participants were invited to the two identical test days, which took place in the morning between 07.00 am and 12.00 am, with similar starting times for both test days, and lasted approximately 2 h. Participants were required to fast overnight (drinking water was permitted). On arrival of the test day, participants provided a blood sample in fasted state, filled out questionnaires, and were then asked to complete three tasks on the computer: two reward learning tasks (results of the reward learning task are reported elsewhere) and a modified version of the Montreal Imaging Stress Task (mMIST). The mMIST consists of 3 segments (baseline, stress exposure and recovery, each segment lasted 5 min). During the mMIST, an ECG was recorded, starting approximately 3 min prior to the baseline of the task, while participants were seated in front of the computer, to allow for some adaptation to the recording system. After the stress task, another blood sample was taken, approximately 45 min after the stress task onset. In addition, before, shortly after, and 20 min after completion of the stress task, cortisol samples were obtained using Salivette^®^ (Sarstedt). We analysed cortisol levels using a standard ELISA; however, because our stress task occurred in the morning, our results showed a decrease across all participants and the stress condition, likely driven by the cortisol awakening response, rendering the findings uninterpretable. Therefore, we do not report cortisol results.

### Participants

Participants were included at the Department of Psychiatry, Psychosomatic Medicine and Psychotherapy at the University Hospital Frankfurt between 24/05/2021 and 27/07/2023. Only clinically diagnosed MDD patients fulfilling the DSM-5 criteria were included in the MDD-group. Potential comorbid diagnoses were assessed with the MINI Diagnostic Interview according to DSM-5 criteria and assessed by trained raters. All participants in the healthy control group had to have an inconspicuous MINI result. All participants were between 18–65 years old. They were excluded if they satisfied the criteria for a bipolar disorder, schizophrenic psychosis, substance abuse disorder or dementia, neurological, autoimmune, rheumatological, neoplastic, dermatologic, or metabolic disorders and for healthy controls only, if they had a previous or current diagnosis of depression. Furthermore, participants with depression induced by substance abuse were excluded. Given that participants with MDD frequently show a high BMI, we excluded only those with a BMI higher than 35 (severe obesity) both for controls and participants with MDD to reflect variability of a naturalistic sample. Participants excluded if they were pregnant or lactating, or had an acute infection as evidenced by CRP levels above 10 mg/L on testday 1 or 2. In case of any respiratory infection, all procedures were postponed until the participant was symptom-free for at least 48 h. For participants with MDD, there was no requirement for treatment resistance.

### Intervention

#### Transcutaneous vagus nerve and sham stimulation

TaVNS was applied at the cymba conchae of the right ear, while sham stimulation was applied with identical parameters at the earlobe (see Fig. 1). Stimulation of the right rather than the left ear was based on preclinical evidence indicating that the right VN has stronger anatomical and functional connections to limbic structures involved in reward and emotional processing [30]. Although both left and right VNS influence cardiac activity, right-sided stimulation has historically been avoided due to the denser innervation of the sinoatrial node by the right VN and a theoretical risk of bradycardia. However, no empirical evidence to date supports safety concerns with transcutaneous right- or bilateral VNS [31] and increases in HRV have been reported following stimulation on both sides. Importantly, in contrast to implanted cervical VNS, taVNS is thought to produce no direct efferent effects on the heart or spleen, sites relevant to cardiac regulation and immune modulation via the cholinergic anti-inflammatory pathway. Therefore, observed physiological changes, including effects on HRV, are likely driven by afferent stimulation of auricular vagal fibers projecting to the brainstem, which then exert downstream influence through central autonomic and neuromodulatory networks. This distinction highlights that taVNS engages brain circuits differently than cervical VNS, where off-target efferent effects cannot be ruled out.

Recent findings further reveal striking lateralization in VN connectivity with the dopaminergic midbrain [31]. Specifically, right cervical VNS appears to engage midbrain dopaminergic pathways more effectively than left-sided stimulation. It has been proposed that right-sided VNS may preferentially activate plasticity-promoting neuromodulatory systems. These findings position right-sided VNS as a promising strategy for enhancing dopamine-dependent processes. Given that one aim of our study was to investigate reward-related processes (reported elsewhere), we opted for right-sided stimulation.

All participants received both interventions (e.g., taVNS either at day 1 or 2 and sham on the other day) in a randomized order. The NEMOS^®^ device for research use (tVNS Technologies, Erlangen) was used, with a pulse duration of 250µs, a pulse frequency of 25 Hz, and a stimulation cycle duration of 32 s on and 28 s off. The stimulation intensity was minimally 100 µA and maximally 5000 µA and was ramped up during the 32 s stimulation cycle. Stimulation intensity was individually determined for each participant and each test day, by increasing the intensity until the participant reported an uncomfortable sensation and then brought down in 100 µA steps until the participant reported a tingling sensation that was not painful. Individual thresholds were recorded. The median stimulation intensity tolerated for sham was 850 µA (range 300–5000 µA) for tVNS it was 800 µA (200–3900 µA). The stimulation started after the blood sample was taken and the baseline assessments (e.g., BDI, demographic questionnaires) had taken place, approximately 10 min before the first reward learning task was initiated. Stimulation was applied continuously for approximately 90 min during the experimental session. Given that stimulation started while participants were filling out questionnaires, slight deviations, corresponding to the time participants required to fill out questionnaires, are possible.

### ECG recording and processing

An electrocardiogram (ECG) was recorded using a 10-channel isolated bioamplifier (NeXus-10 MKII, Mind Media BV, Netherlands). Three disposable electrodes (Kendall™, H66LG electrodes) in a modified lead II configuration were placed on the chest to record the ECG at a sampling frequency of 1024 Hz. BioTrace + Software (Mind Media BV, Netherlands) was used to check the signal and export the data. For the modified lead II recording electrodes were placed on the right and left upper chest (approximating the arms), and the ground electrode was placed on the lower left abdomen. ECG signal processing was performed with Kubios Pro Scientific Analysis software [32], which has an inbuilt R peak detection and inbuilt artifact and noise correction. A visual inspection of raw data was performed per segment of interest (the 5-min baseline, stress and recovery). Noise detection was used with the standard settings, and unclear beats were removed manually. Segments with more than 5% of beats in need of correction were excluded from the analysis. For the present analysis we extracted heart rate (HR) in beats per minute, root mean square of successive differences between normal heartbeats (RMSSD) in ms, the absolute power of high frequency heartrate variability (HF)-HRV (0.15–0.4 Hz), and low frequency (LF)-HRV (0.04–0.15 Hz) both fast Fourier transformed. We opted for these measures given our interest in cardiac vagal modulation, which can be approximated by RMSSD. For the sake of comparability with other articles reporting frequency-domain rather than time-domain measures, we also report the HF-HRV. Furthermore, for completeness, we also report LF-HRV, and HR was selected as a general index of cardiac output and physiological homeostasis [33].

### Stratification based on RMSSD

After conducting the analysis separated by diagnosis (MDD vs. HC), we performed a post-hoc stratification based on the biological parameter RMSSD. To stratify the participants, we used a median split for RMSSD in milliseconds (ms) across all participants to obtain baseline RMSSD groups. We used the 5 min baseline of the sham intervention for stratification purposes. The RMSSD median used for stratification was 39.14 ms (for the low RMSSD group the range was 5.51 ms - 38.75 ms and for the high group 39.14 ms - 150.80 ms). If no data was present for this timepoint, missing values for RMSSD (n = 11) were imputed using all ECG data with chained random forests as implemented in the *missRanger* package [34] in R. We opted for RMSSD instead of HF-HRV or respiratory sinus arrhythmia because it is slightly less dependent on respiration [35] and respiration was not measured in this study. Given the known influence of tricyclic antidepressants (TCA) on HRV, we excluded three participants on TCA in the analysis stratified by RMSSD group. The results in- and excluding participants on TCA for both stratification approaches (1. stratification based on diagnosis, and 2. stratification based on RMSSD group) are provided in the supplementary tables.

### Blood sample preprocessing and analysis

Blood samples of the fasted participants were collected by venipuncture upon arrival and 45 min after the stress task. The blood draw was performed in a fasted state to reduce variability for the inflammatory protein analyses, given that postprandial levels of proteins can differ, and to standardize metabolic states between testing days [36, 37]. Blood was collected in K3EDTA Monvettes (S-Monovette® K3 EDTA) and immediately transferred to cooling containers. The samples were then spun down (2000g, 10 min) in a precooled centrifuge (4 °C) no later than 30 min after blood sampling. The resulting plasma was then aliquoted into Eppendorf tubes and stored at −70 °C until further analysis. To obtain levels of high sensitive (hs) IL-6 and hs TNF-α, we used enzyme-linked immunosorbent assay with precoated kits (IL-6: Human IL-6 High Sensitivity ELISA, Art.Nr.: BMS213HS; TNF-α: Human TNF-α High Sensitivity ELISA Kit, Art.Nr.: BMS223HS and BMS223-2HS both, Invitrogen/ThermoFisher Scientific) as per manufacturer’s instructions. Samples were measured in duplicate, blank values were subtracted, and a standard curve was obtained for each plate using Microsoft excel, optical densities were converted to concentrations in pg/mL. If the sample duplicate’s coefficient of variation deviated more than 15%, they were repeated. Values below the detection level were replaced by the level of detection (LOD) divided by 2. For TNF-α analytical sensitivity was 0.13 pg/mL, for IL-6 0.03 pg/ml. For TNF-α, of the 361 measurements, 47 were below level of detection and were replaced by the LOD divided by 2 (i.e., 0.13/2). No levels were below the detection level for IL-6. Concentrations ranged from 0.35 pg/mL - 55.70 pg/mL for IL6, and from 0 (undetectable levels) - 8.37 pg/mL for TNF-α.

### Sample size

The sample size was determined with a predefined power calculation, for a repeated measures Analysis of Variance (ANOVA) with a within and between effects interaction. A sample size of 76 was determined, given an assumed effect size of 0.25, alpha 0.05, and a reasonable power of 0.95, with a correlation among measures of r = 0.3, 2 groups and 2 measurements (i.e., taVNS/sham) (calculated with G*Power 3.1.9.6). Given the potential dropout effects, and assuming that around 20% of values detected with ELISAs are below the threshold of detection, we aimed to recruit 100 participants at T1 (with 1:1 group distributions). A simulation analysis showed that with 100 participants, a smaller effect size of just below f = 0.152 would still be detected with 80% power (see Supplementary Fig. 1).

### Randomization

Participants were randomized to receive either sham stimulation or verum stimulation based on a pre-established list, composed by a random sequence in Excel. Participant IDs were linked to the allocation and could not be changed. The list was assembled in three blocks of 50 participants. Researchers were not blinded to the allocation, but participants were blinded, i.e., they were not informed of which intervention they received on each day. The random allocation sequence was generated by the senior scientists, while allocation was performed by the study staff. The procedure of sham stimulation and taVNS were identical, except for the location of stimulation. Care was taken to spend an equal amount of time on the establishment of the stimulation intensity thresholds and experimental procedures in both intervention arms.

### Questionnaires

The Mini-Neuropsychiatric International Interview (MINI, version 7.0.2 for DSM-5) [38] was assessed to exclude psychiatric comorbidities. The MINI is a short, well-validated, semi-structured diagnostic interview for major psychiatric disorders. Items refer to the last month and/or the entire life and are rated on a yes/no basis, allowing us to assess the absence or presence of psychiatric disorders, based on DSM-5 criteria. The Montgomery Asberg Depression Rating Scale (MADRS) is a well-validated 10-item clinical interview assessing symptoms of depression in the past 7 days, with a cumulative score indicative of the presence of no, light, moderate, or severe depression [39]. Proposed cut-offs are 0–6 (absence of depression), 7–19 (mild depression), 20–34 (moderate depression) and 35–60 (severe depression). In addition, the self-rated Beck’s Depression Inventory (BDI-II) and the Perceived Stress Scale (PSS) [40] were administered. The BDI-II is a 21-item, self-rated scale for depressive symptoms with good sensitivity and specificity [41]. The PSS-10 is a commonly used 10-item scale to assess perceived stress in the past 4 weeks. Scores range from 0 (no stress) to 40 (high stress). The Childhood Trauma Questionnaire (CTQ) was collected to assess childhood trauma [42].

### Visual Analogue Scales

After the baseline, after the stress and after the recovery phases, participants rated eleven items on a 100-mm Visual Analogue Scale (VAS). Two composite scores were calculated for the items of the VAS scale (one containing negative emotions, the other one positive emotions, consisting of the sum of the items (positive: energetic, motivated, happy, in control; negative: scared, alert, stressed, powerless, tense. Additionally, given the experimental stress exposure, we explored the answers on the VAS item “stressed” separately, hereafter named “stress perception”.

### Experimental stress task

To induce stress, we used an adapted, customized, and computerized variation of the mental arithmetic stress task similar to the Montreal Stress Imaging task (MIST) [43]. The task includes a 5 min baseline, a 1 min-control/demonstration task (i.e., a mental arithmetic without stressful elements), a 5min mental arithmetic task with stressful elements (stress phase), and a 5min recovery phase. In the current version, the following elements are included to induce stress: social comparison (e.g., playing against an “imaginative peer” whose progress is monitored by a progress bar on the right side of the screen), adaptive time pressure (i.e., if the participants answered correctly, they would receive less time to answer the next question; if they answered wrong several times, the time limit was increased) and feedback by the researcher (i.e., two times verbal feedback to “answer faster” or “try to answer correctly”). This version is a computerized task, which requires participants to select the right answer on the computer screen without requirement for verbalization and participants were instructed not to talk in order to not disrupt breathing patterns.

### Statistical methods

Demographic variables between groups were compared using t-tests, the Wilcoxon test (W), or the Chi-square (χ^2^) test for proportions. Model assumptions were checked, and if residuals were not normally distributed, variables were transformed as indicated. To assess the VAS score, a composite score was calculated for a negative and a positive component, by summing up relevant items. To assess our main hypothesis, we fitted a linear mixed model (estimated using REML and if necessary nloptwrap optimizer) to predict variables of interest with Intervention (categorical, taVNS vs. sham), RMSSD (categorical, high vs. low RMSSD group; or continuous RMSSD, see Supplementary Tables), Timepoint (categorical, baseline, stress, recovery), Group (categorical, MDD vs HC), Sex (categorical), Age (continuous), Testday (categorical, day 1 vs day 2) and Stimulation Intensity (continuous) (for the analysis comparing MDD vs HC: formula: DV~ Intervention * Group * Timepoint *+* *Sex* *+* *Age* *+* *Testday* *+* *Stimulation Intensity*; for the analysis by RMSSD: DV~ Intervention * RMSSD * Timepoint *+* *Sex* *+* *Age* *+* *Testday* *+* *Stimulation Intensity*). Testday was added to the model in order to control for any habituation effects to the task. The model included ID as a random effect (*formula*: *~1* | *ID*). Models were fitted using *lme4* [44] and *lmerTest* packages [45]. Furthermore, for HRV, we also conducted a model including an Age*Intervention interaction reported separately. A type-III ANOVA table was computed for F and p values for fixed effects using Satterthwaite’s method for the approximation to degrees of freedom. We repeated the same model for post-hoc stratified analyses, where instead of diagnostic group, we used RMSSD-group (low vs. high). The insignificant 3-way interaction for RMSSD was compared with post-hoc tests, given the specific interest to our study design. Where reported, post-hoc tests were computed as contrasts with the *emmeans* package [46] and if applicable to the contrasts, the standard inbuilt Tukey method for comparing a family of tests of the *emmeans* package was automatically applied. Additionally, we also investigated antidepressant medication status yes/no (here, most participants were taking SSRIs or SNRIs). Results did not change, and antidepressant medication was not a significant predictor for HRV at the exception of LF-HRV (see Supplementary Information) therefore the models are reported without antidepressant intake as covariate. An overview of the association between antidepressant medication class (none, SSRI, SNRI, TCA, other) and baseline HRV parameters can be found in Supplementary Table 1D).

All statistical analyses were performed with RStudio (Version 4.3.1.) and graphical representations were made in Graphpad Prism, based on summary data extracted from R [47].

## Results

**Fig. 2:**
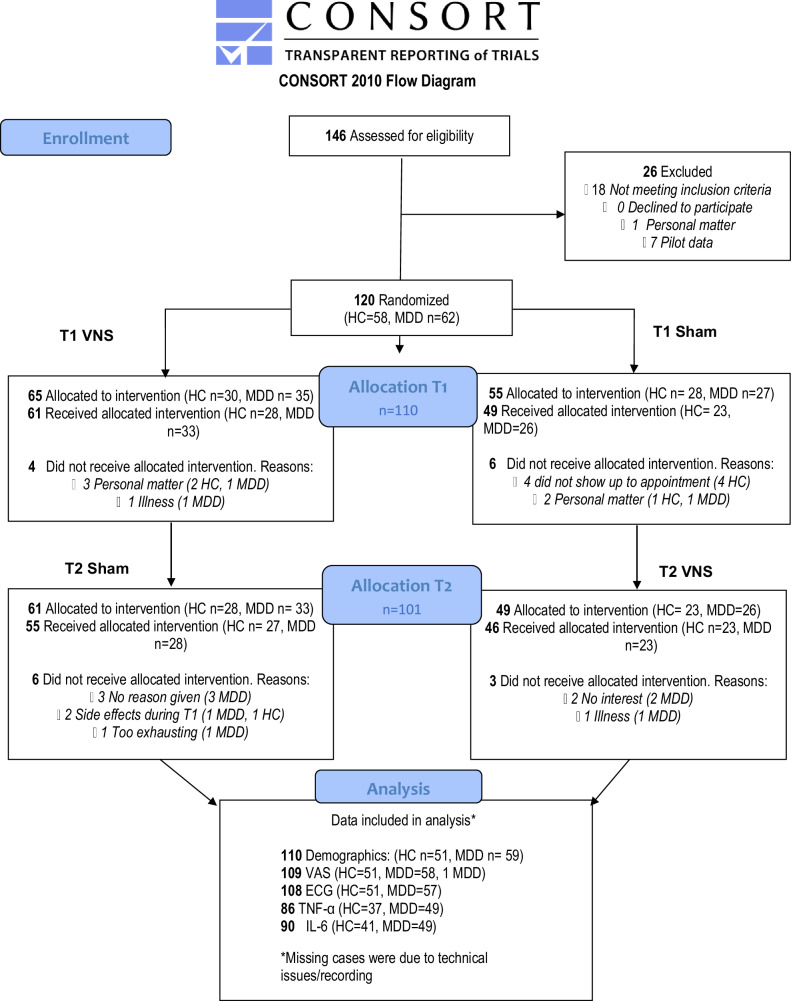
Consort Flow Diagram of the MODULATE study. Ninety-four percent (61/65) of the randomized participants completed a first test day (T1) with taVNS versus 89 percent (49/55) with a sham stimulation; the difference was not significant (p = 0.543). At the second test day (T2) 84.61 (55/65) of randomized participants received the planned sham stimulation and 83.64% (46/55) received the planned verum stimulation (p > 0.999). In total, 51 controls and 59 participants with MDD were randomized to the intervention and completed the first session (T1) and were thus used for analysis.

In the period from May 2021 to August 2023, 146 participants were assessed for eligibility, of which 26 were excluded or piloted with a different study design, 120 were randomized, 110 completed their first intervention and 101 participants completed their second cross-over phase (Fig. 2). The trial was ended after the target of included participants was reached.

### Baseline data

For all presented analyses, we compared participants with MDD versus healthy controls (HC) (Table 1). Participants with MDD were older compared to the control group, but had a similar BMI, sex, and smoking status distribution. There was no difference between education level or contraception intake between groups. All questionnaires (BDI-II, MADRS CTQ, PSS) differed significantly.

**Table 1:** Demographic Characteristics of Participants by Diagnosis.

	N	CTRL, N = 51^*1*^	MDD, N = 59^*1*^	p-value^*2*^
Sample characteristics				
Age	109	30.24 (10.78)	38.79 (13.29)	**<0.001**
Sex	110			0.8
Female		33 (65%)	37 (63%)	
Male		18 (35%)	22 (37%)	
BMI	109	23.57 (2.59)	24.32 (3.82)	0.2
Smoker	110			0.5
No		43 (84%)	44 (75%)	
Occasional		4 (7.8%)	5 (8.5%)	
Yes		4 (7.8%)	9 (15%)	
Unknown		0 (0%)	1 (1.7%)	
Contraception	109			0.2
no		19 (37%)	23 (40%)	
yes		11 (22%)	5 (8.6%)	
postmenopausal		1 (2.0%)	6 (10%)	
male		18 (35%)	22 (38%)	
unknown		2 (3.9%)	2 (3.4%)	
Education	110			0.5
0- no degree		0 (0%)	2 (3.4%)	
1- primary school		0 (0%)	1 (1.7%)	
2- secondary school		20 (39%)	16 (27%)	
3- secondary education		6 (12%)	7 (12%)	
4- university degree		24 (47%)	32 (54%)	
5- doctoral degree		1 (2.0%)	0 (0%)	
unknown		0 (0%)	1 (1.7%)	
Unmedicated	109			**<0.001**
no		3 (5.9%)	38 (66%)	
yes		48 (94%)	17 (29%)	
nr		0 (0%)	3 (5.2%)	
Antidepressants	109			**<0.001**
no		51 (100%)	20 (34%)	
yes		0 (0%)	34 (59%)	
nr		0 (0%)	4 (6.9%)	
Questionnaires				
BDI-II	109	1.31 (2.16)	24.86 (10.28)	**<0.001**
MADRS (SD, range)	110	1.20 (2.18, 0–9)	25.66 (8.66, 7–48)	**<0.001**
CTQ Sum Score	108	34.20 (10.51)	51.19 (18.31)	**<0.001**
PSS-10	109	10.37 (4.69)	25.03 (5.62)	**<0.001**

^*1*^Mean (SD); n (%).

^*2*^Wilcoxon rank sum test; Pearson’s Chi-squared test; Fisher’s exact test.

*CTRL* control group, *MDD* major depressive disorder group. Bold values indicate significant results.*nr* not reported.

### Results of blinding

Most participants in both intervention arms (sham and taVNS) believed that they were receiving the taVNS stimulation (84% of all cases, 81% during sham, 86% during taVNS X^2^ = 1.00, p = 0.317) and stimulation intensity was comparable for both interventions (sham median: 850 µA, tVNS median: 800 µA, Wilcoxon test (W) = 5957.5, p = 0.094).

### Planned analysis

#### Diagnosis was not associated with effect of taVNS

To assess if the efficacy of taVNS on our outcomes was modified by diagnosis, we conducted analyses comparing the two groups (MDD vs. HC), adjusted for covariates (see Supplementary Table 1). Timepoint had a significant effect (p < 0.001) on subjective stress levels, which increased during the stress task and reduced following recovery. We found a main effect of group (p < 0.001): Participants with MDD reported higher levels of stress compared to healthy controls across all time points. For emotions, stress perception and HRV, we found a significant main effect of group and timepoint, but no main effect for intervention nor for the interaction between diagnosis and intervention (taVNS vs. sham * MDD vs HC, see Table 2 for the main effect of Intervention and the Intervention*Group interactions). Full models can be found in Supplementary Table 1. Due to the absence of intervention effects, in the following, we decided to apply a post-hoc stratification based on RMSSD group instead of diagnosis, to assess whether those with lower RMSSD would profit more from taVNS than those with higher RMSSD, regardless of diagnosis. Of note, we opted for a median split because we believe that it has clinical utility but also performed analyses with RMSSD as a continuous predictor, which yielded comparable and highly significant results, and is reported in the supplementary material.

**Table 2:** Results of Planned analysis comparing MDD and HC.

		Intervention	Group* Intervention		
		F	p	F	p
**Emotions**	Positive [Composite score]	1.74	0.187	0.09	0.768
	Negative	1.18	0.278	0.34	0.561
	[Composite score]				
	Stress [%]	0.62	0.431	1.26	0.263
**HRV**	Heart Rate	0.35	0.552	0.45	0.501
	RMSSD	1.75	0.187	0.06	0.809
	HF-HRV	0.59	0.444	0.05	0.819
	LF-HRV	1.28	0.259	1.44	0.231
	LF/HF ratio	0.19	0.66	1.98	0.16
**Inflammation**	IL-6 in pg/ml	1.12	0.291	3.04	0.082
	TNF-α in pg/mL	0.57	0.45	0.01	0.922

No significant effects of intervention, or intervention *group were found.

Variables were transformed using log or Tukey’s ladder of power transformations and models included covariates as well as timepoint and interaction effects. Full models and models excluding those taking TCA can be found in the supplementary material Table 2.

### Demographics based on RMSSD group

When split by RMSSD, 55 participants were in the high and 54 in the low RMSSD group. In the low RMSSD group, 39 of 54 (72%) were MDD participants, in the high RMSSD group, 19 of 55 (35%) were MDD participants (see Supplementary Table 2A for demographics by RMSSD). The low RMSSD group had higher depression scores and higher chronic stress levels, which are likely attributable to the higher percentage of participants with MDD in the low RMSSD group (Supplementary Table 2B and C), but childhood trauma was also higher in the low RMSSD control group (p = 0.033), see Supplementary Table 2B. Baseline RMSSD during sham as continuous variable correlated negatively with MADRS values (rho = −0.33, p < 0.001), but not with TNF-α or IL-6 (IL-6: rho = −1.08, p = 0.313, TNF: rho = −0.081, p = 0.458).

### The impact of taVNS on stress perception depends on RMSSD

The interaction of intervention (taVNS vs. sham) and RMSSD group was significant (p = 0.024). Follow-up tests showed that for those with high RMSSD, taVNS did not affect stress levels (t = 0.06, p = 0.549), but significantly increased stress perception in those with low RMSSD (t = −2.63, p = 0.009). No other interactions were significant.

**Fig. 3:**
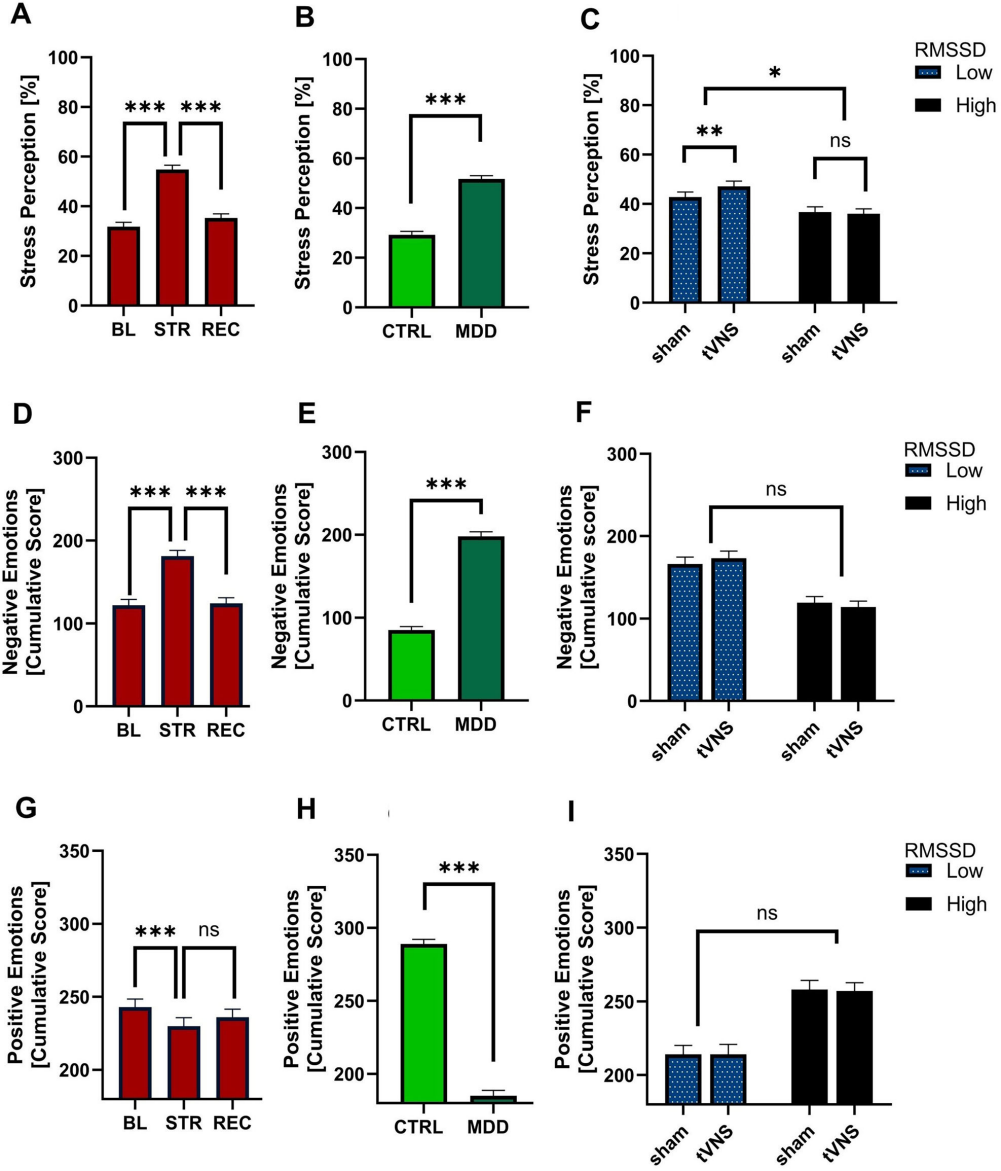
Impact of acute stress and taVNS on emotional reactivity. **A-C** Effects on Stress Perception. All values were transformed with Tukey ladder of power transformation for statistical analyses but are presented as untransformed scores. From left to right: **A**. Main effect of acute stress, **B**. Main effect of Group, **C**. 2-way interaction effect of intervention and RMSSD group. **D-F** Effects on Negative Emotions (Composite Score). From left to right: **D**. Main effect of acute stress, **E**. Main effect of Group, **F**. 2-way interaction effect of intervention and RMSSD group, **G-I** Effects on Positive Emotions (Composite Score). From left to right: **G**. Main effect of acute stress, **H**. Main effect of Group, **I**. 2-way interaction effect of intervention and RMSSD group, Significance:***:p < 0.001, ** p < 0.01, *p < 0.05, #p < 0.1, ns:p > 0.1.

Timepoint had a significant main effect for negative emotions (p < 0.001), but not for positive emotions(p = 0.676). The main effect of group was significant in both models (both p < 0.001), but neither the grouping by RMSSD, nor the intervention had an effect on either emotion (see Supplementary Table 3A for fixed effect estimates of the full models and Fig. 3A-I. for graphical representations). Analyses with RMSSD as a continuous score yielded similar results (Supplementary Table 3B).

### The impact of taVNS on heart rate variability depends on RMSSD

To assess the impact of RMSSD group and taVNS on the autonomic stress response, we investigated RMSSD and other measures of heart rate variability (HR, HF-HRV, LF-HRV and LF/HF ratio).

Main effects for timepoint and RMSSD group were significant (all p < 0.05) for all measures, only for the LF-HF ratio, timepoint was not significant (p = 0.149), see Supplementary Table 4A. We found an interaction of RMSSD group and intervention for RMSSD (p < 0.001), HF-HRV (p = 0.002), LF-HRV (p = 0.026), but not HR (p = 0.121) or LF/HF ratio (p = 0.127) (see Supplementary Table 4B for post-hoc comparisons of interactions).

**Fig. 4:**
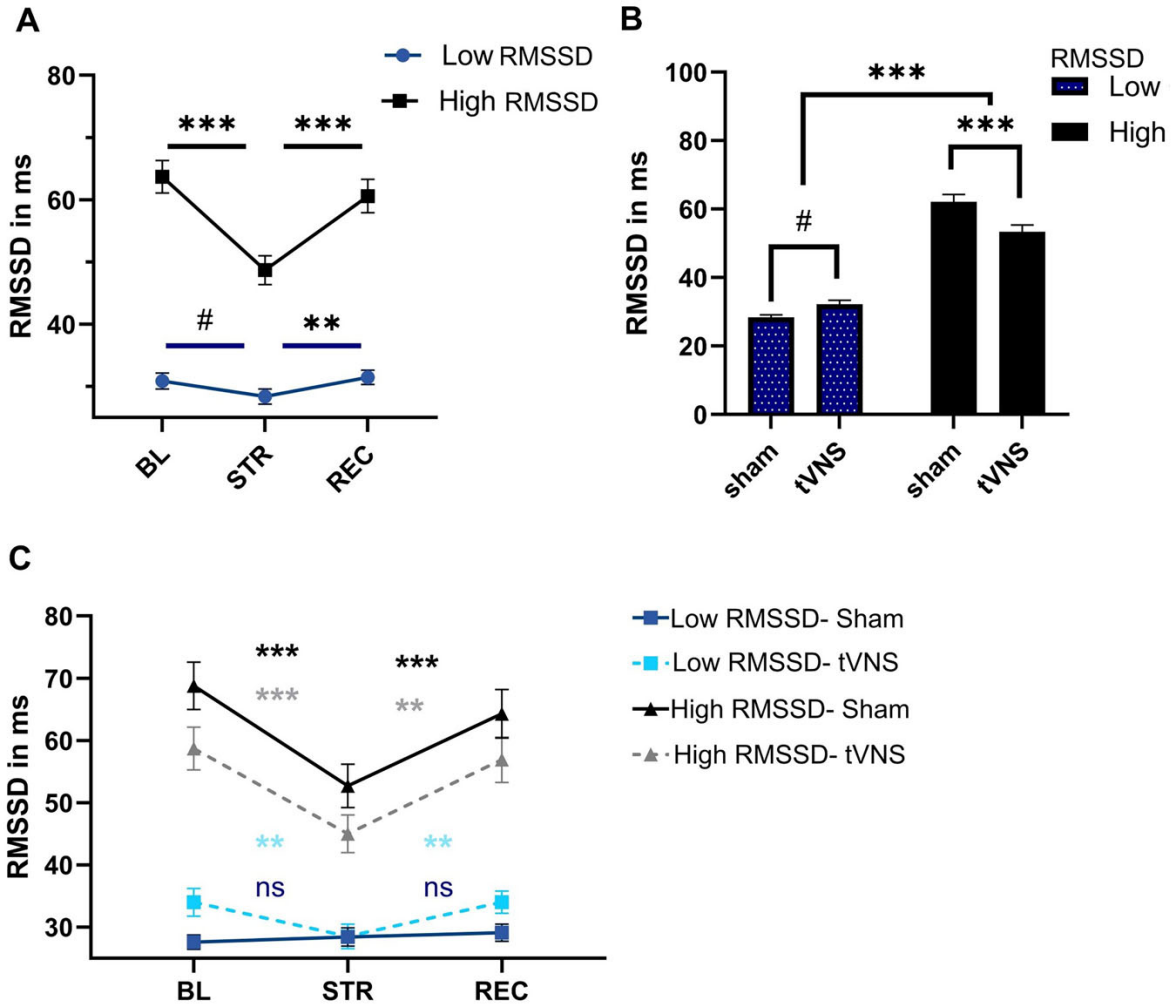
Impact of acute stress and taVNS on heart rate variabitlity (RMSSD in ms). Results are presented as raw values (in ms). **A** Interaction of Stress and RMSSD group; **B** Interaction of intervention with RMSSD group. **C** 3-way interaction for Stress, Intervention and RMSSD group. Note that scales differ between (**A,B** and **C**), to be able to display all error bars appropriately. Significance:***:p < 0.001, ** p < 0.01, *p < 0.05, #p < 0.1, ns:p > 0.1.

Follow-up tests showed that RMSSD and HF-HRV numerically increased in the low RMSSD group during taVNS (sham-taVNS; RMSSD: t = −1.96, p = 0.050, HF-HRV t = −1.84, p = 0.067), but taVNS reduced RMSSD and HF-HRV in the high RMSSD group (sham-taVNS; RMSSD t = 3.58, p < 0.001; HF-HRV: t = 2.79, p = 0.006), see Fig. 4B. Furthermore, for all variables, except LF-HRV and LF/HF ratio, a significant RMSSD*timepoint interaction occurred (all P < 0.05, see Supplementary Table 4A and 4B). Post-hoc tests for RMSSD showed that participants in the low RMSSD group did not react to stress (baseline-stress: RMSSD: t = 2.23, p = 0.067), compared to strong reactivity in the high RMSSD group (baseline-stress: RMSSD: t = 7.08, p < 0.001), while both had significant recovery, albeit numerically stronger in the high RMSSD group (high: t = −5.15 vs. low: t = −2.98), see Fig. 4A. Results with RMSSD used as a continuous predictor were comparable to the analysis using a factor for HF-HRV and RMSSD (see Supplementary Table 4C for statistics).

Furthermore, given the broad age range of participants (18–65), we tested whether taVNS effects on HR and HRV were moderated by age. Significant intervention*age interactions were observed for HF-HRV and RMSSD (Supplementary Table 4D). Estimated marginal means revealed clear age-dependent effects for RMSSD and HF-HRV: at age 20, RMSSD/HF-HRV were higher under taVNS than sham, whereas at ages 40–60, higher values were observed in the sham condition (Supplementary Table 4E). Importantly, in models including the intervention*age interaction, the intervention*RMSSD interaction remained significant, indicating that, although age modulates responsiveness, taVNS effects are also robustly influenced by individual baseline RMSSD rather than being solely driven by age differences.

### Exploratory post-hoc analyses 3-way interaction for RMSSD shows recovery of blunted stress response

Given our interest in the treatment effect, we also conducted exploratory contrasts for the non-significant three-way interaction of RMSSD. Contrasts showed that while during sham stimulation, there was indeed a blunted reactivity and recovery for RMSSD in the low RMSSD group (sham; baseline-stress: t = 0.19, p = 0.981, stress-recovery: t = −0.82, p = 0.692), this was restored during taVNS (baseline-stress: t = 2.96, p = 0.009, stress-recovery: t = −3.39, p = 0.002). In the high RMSSD group both sham and taVNS showed significant reactivity and recovery during sham (sham; baseline-stress: t = 4.79, p < 0.001, stress-recovery: t = −3.52, p = 0.001) and taVNS (baseline-stress: t = 5.25, p < 0.001, stress-recovery: t = −4.29, p < 0.001), see Fig. 4C. It should be noted that this analysis was exploratory in nature and should be interpreted with caution, as our sample size may have been too low to detect reliable effects.

### Effect of taVNS on inflammatory proteins is opposite dependent on RMSSD

**Fig. 5:**
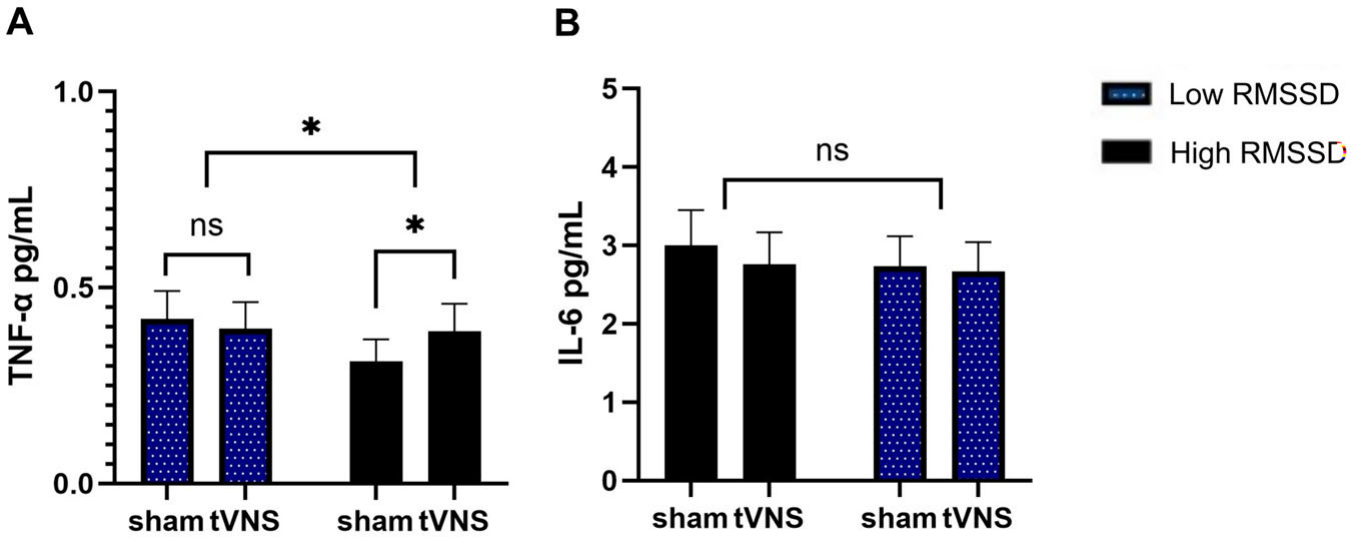
Levels of TNF-α and IL-6 according to intervention. Values are back-transformed and were measured in pg/mL. They represent the averaged effect of RMSSD group*Intervention regardless of condition (BL/STR). **A**. Intervention*RMSSD(low/high) for TNF-α**, B**. Intervention*RMSSD(low/high) for IL-6. Significance: ***:p < 0.001, ** p < 0.01, *p < 0.05, #p < 0.1, ns:p > 0.1.

Overall, as expected in a population without overt inflammatory conditions, levels of IL-6 and TNF-α were low. No main effects of RMSSD group, or timepoint occurred for IL-6 or TNF-α (see Supplementary Table 5A for fixed effects). For TNF-α, an intervention*RMSSD group interaction occurred (p = 0.038). Post-hoc comparisons showed that regardless of group or timepoint, in those with higher RMSSD, TNF-α was significantly *higher* during taVNS than during sham stimulation (sham-taVNS: t = −2.09, p = 0.038) while in those with low RMSSD, TNF-α was not significantly altered (sham-taVNS: t = 0.70, p = 0.483). See Fig. 5 for an overview. If RMSSD (sham, baseline) was used as a continuous variable, the interaction of RMSSD*Intervention was not significant for TNF-α (p = 0.232), see Supplementary Table 5B.

## Discussion

In this counter-balanced, randomized-controlled study, we aimed to assess whether taVNS could improve the acute stress response in participants with MDD by improving subjective stress perception, enhancing cardiac reactivity and reducing inflammation compared to healthy controls. Contrary to our hypothesis, we found no effect of taVNS on these outcomes, nor any interaction of taVNS and diagnosis. In other words, taVNS had no significant impact on our outcomes, with no difference between MDD participants and HC.

Subsequently, we decided to use post-hoc stratification by a biological marker, RMSSD, to approximate cardiac vagal modulation (with RMSSD as a surrogate marker). We reasoned that participants with low RMSSD, indicating low VN activity, were most likely to profit from taVNS. In the low RMSSD group (regardless of diagnosis), taVNS improved the previously absent cardiac vagal reactivity to stress, increased the perception of stress, but only had a minor, non-significant reductive effect on TNF-α. In contrast, and unexpectedly, if RMSSD was high at baseline, taVNS *dampened* heart rate variability (HRV), *in*creased HR and significantly *in*creased levels of TNF-α. Furthermore, for HRV-based measures, taVNS had a different effect in younger compared to older participants. Our findings clearly suggest that the effects of taVNS depend on baseline RMSSD and age, suggesting that consideration of both is essential for future studies.

### taVNS: No effect on biological outcomes in MDD?

An adequate response to acute stress is fundamental for survival and is tightly regulated by the autonomic nervous system. Persisting exposure to physical or emotional stress, however, can disrupt homeostasis, mitigate basal cardiac parasympathetic activity, and impair the physiological ability to face sudden challenges, as often observed in participants suffering from MDD. VNS provides a targeted approach to directly stimulate the underlying biological pathways that are impaired by chronic stress exposure. Surprisingly, against our hypotheses, there was an absence of a main and/or interaction effects for the intervention in our primary analyses comparing participants with MDD and controls. This could indicate that the non-invasive form of taVNS does not have any acute effects, even in a relatively large sample such as ours, or, that acute stimulation sessions are insufficient to produce these effects. However, our post-hoc stratification revealed a critical feature linked to the response to taVNS: the individual RMSSD at baseline. Only in the low RMSSD group, taVNS improved stress perception and recovered cardiac reactivity. While we expected the strongest effects of taVNS for those with low RMSSD, and somewhat attenuated effects in those with high RMSSD, this was not the case. Contrary to our expectations, we did not find weaker effects in those with high RMSSD, but instead, taVNS had stronger and *opposed* effects to the anti-inflammatory, HRV-enhancing effects we expected from the literature. This is a crucial insight, as effects in opposed directions in those with high and low RMSSD may lead to null findings for (ta)VNS paradigms, if baseline levels are disregarded.

The absence of a significant modifying effect of MDD on the intervention may be partly attributed to the diagnostic criteria for depression, which currently rely solely on symptomatology and likely encompass various distinct pathophysiological mechanisms. This may explain why the majority, but not all, MDD participants exhibit low RMSSD in our study, which is in line with recent meta-analytic findings [48]. Consequently, RMSSD could serve as a valuable tool for selecting participants based on a biological stratification marker for VNS treatment.

### Chronic stress is linked to RMSSD levels

In line with previous research [49], we found that chronic stress and childhood trauma were associated with low RMSSD in our sample. This association has been predominantly reported in clinical populations suffering from chronic stress conditions (i.e., Posttraumatic Stress Disorder, Anxiety Disorders, MDD) [50]. The well-orchestrated response to acute stress starts with an increase in sympathetic activity, catecholamine and cortisol production, energy supply through gluconeogenesis and lipolysis, and cardiac (e.g. increased heart rate and blood pressure) and immune arousal, but is temporally limited and terminated by parasympathetic activation. Yet, sustained stress and the lack of habituation to stressors can lead to deficient RMSSD (i.e., [51]), failing to regulate the aforementioned systems and therefore leading to a chronic elevation of sympathetic activity, catecholamine, and cortisol production and reduced cardiac vagal modulation. As in previous studies [5, 52, 53], we here observed the biological effects of chronic stress often described in the literature: We found that in the low RMSSD group, the baseline level of HR was higher, cardiac stress reactivity was blunted, and baseline levels of perceived stress and depressive symptoms were higher.

### Differential effects of taVNS depend on RMSSD: mechanistic considerations

Intriguingly, taVNS influenced cardiac reactivity and recovery for those with low RMSSD. Stimulation of the VN’s sensory afferents at the outer ear is considered to activate the dorsomotor nucleus. This activation relayed through the afferent nucleus (NTS) ^54^, modulates the parasympathetic projections directed towards the sinoatrial and atrioventricular nodal cells as well as the atrial myocytes [54, 55]. It is thus possible that taVNS can counteract the effects of chronic stress by activating parasympathetic projections. The opposed effects in the high RMSSD group suggest that this mechanism may differ in this group, either by engagement of a different mechanism, overactivation of the projections and/or by activation of the sympathetic rather than parasympathetic nervous system. Indeed, previous studies have discussed the possibility of low-level sympatho-excitation by vagal afferents due to bioelectric stimulation as applied during taVNS [56]. This could provide a potential explanation for the inconsistency reported regarding (ta)VNS effects on heart rate variability, which have shown reductions [57–59], increases [60–63], or no distinguishable effect [64–67], summing up to a lack of a consistent effect on HRV [68].

### Age moderates the effect of taVNS for HRV-based measured

Another intriguing finding was the significant interaction between age and our intervention. Particularly for RMSSD, older participants showed a reduction during taVNS, whereas younger participants showed either increases or no change. This suggests that parasympathetic responsiveness to acute taVNS declines with age, potentially reflecting age-related changes in autonomic flexibility or vagal nerve sensitivity. This finding is in line with consistent evidence showing that HRV and autonomic flexibility decline with age [16, 69]. However, the impact of age on cardiac responses to taVNS has not been systematically examined and warrants further investigation.

### No acute effects of taVNS on emotional states

Furthermore, as a treatment for depression, the integration between the autonomic state, interoceptive signals, and emotional states is crucial and is mediated by highly interconnected brain stem nuclei and higher cortical regions that receive vagal afferents. Stimulation of the vagus nerve (direct and/or via taVNS) provides an intriguing option to investigate the effect of stimulation on this integrative process since it can stimulate the *nucleus tractus solitarii*, and the interconnected limbic and cortical areas that belong to the central autonomic network in both rodents and humans [10–14]. Chronic VNS has been shown to improve negative mood states in humans [2, 3, 70] and low RMSSD has been linked to negative emotional states, in line with our data. A recent rodent study elegantly demonstrated that reduced RMSSD due to prolonged stress resulted in alterations in the connection between the prefrontal cortex and amygdala, leading to anxious behavior similar to that seen in vagotomy, and chronic VNS restored brain signals and the anxious phenotype [51]. In our sample, however, no intervention effect was found for negative emotions after the short intervention period. This is not entirely surprising, as our study was designed to assess the acute mechanistic effects of VNS, and not the anti-depressant effects of VNS, which would require repeated exposure to stimulation. It is thus possible that long-term stimulation would yield different results. Future studies in humans need to shed more light on this process using prolonged exposure.

### Differential effects of taVNS on inflammatory cytokines depending on RMSSD

The seminal discovery by Borovikova et al. [8] highlighted the relevance of the VN for regulating inflammatory responses through the cholinergic anti-inflammatory pathway. Since then, several studies, also in humans, have shown that low cardiac parasympathetic activity is associated with increased cytokines at baseline and after immunological challenges [71, 72]. Selective stimulation of the VN - both invasive and transcutaneous - suppresses circulating cytokines, but most of the significant effects were only applicable to subgroups and in conditions of acute immunological events [23]. Our results showing differential effects of taVNS depending on baseline RMSSD raise new questions: first, our results suggest that taVNS could have a modest anti-inflammatory potential *if* baseline RMSSD is low, whereas if the RMSSD is already high, taVNS can increase TNF-α. Second, we did not find a change in IL-6 after taVNS, which is in line with preclinical literature and suggests a rather specific effect of (ta)VNS on TNF-α. Analog to the findings in HRV, this might explain the rise in cytokines reported following taVNS in healthy human participants (e.g., [73, 74]), who were not recruited based on baseline RMSSD and might have had high RMSSD. Finally, for those in the low RMSSD group, stress perception increased rather than decreased - a finding that is rather counter-intuitive, since successful taVNS is thought to reduce stress. A possible explanation could be that taVNS may increase the awareness of bodily states (i.e., interoception) [75] including the subjective perception of stress, but further research is needed on this topic. We are intrigued whether similar findings can be found in studies using longer durations and different pathologies.

### Inverse effects of taVNS with higher RMSSD? Clinical implications and call for stratification in existing cohorts using (ta)VNS

Although there is a general correlation between high RMSSD, favourable health outcomes and a well-regulated autonomic nervous system, taken together our results suggest that RMSSD beyond a certain threshold, triggers an alternative mechanism (potentially SNS rather than PNS activation) in response to taVNS. Our results align with prior research that documented decreased heart rate variability following (ta)VNS [58, 59] and increases in cytokines in healthy participants [73]. Most of these results involved youthful, healthy participants with probably high RMSSD at baseline, who may have surpassed a threshold with (ta)VNS. In the clinical context, vagal hyperactivity (which we don’t see in our participants) has been linked to bradycardia, syncopes, hypotension, and excessive gastric acid secretion [76–78]. Here, we used post-hoc stratification with a median split for RMSSD as an index of cardiac vagal activation, due to the sample size constraints, but the exploratory analyses using continuous RMSSD produced similar results. It is thus possible that a dose-response relationship is present, which should be explored in larger sample sizes.

We identified one small clinical trial which also used stratification by baseline RMSSD after a long-term intervention with invasive VNS in participants suffering from Crohn’s disease [79]. Intriguingly, the effect of VNS on heart rate variability was restorative in 4 of 5 participants with low RMSSD but decreased in the one person with high RMSSD at baseline. Others have reported that responders to VNS (as defined by more than 20% decrease in LF/HF), had significantly lower basal RMSSD than non-responders [63]. Although it is obvious, that VNS should be particularly beneficial if RMSSD is compromised, the majority of studies so far have not used RMSSD for stratification. From a clinical perspective, if RMSSD is indeed found to be a useful predictor of long-term treatment outcomes of taVNS in other studies, this could contribute strongly to a precision medicine approach beyond psychiatry, reducing waiting times for procedures and permitting clinicians a better prediction of success rates. This is particularly relevant given that between initiation of VNS and clinical response, there can be a time lapse of 3–12 months and cumulative response rates show response rates only for approximately half the population [80].

If HRV data is available, we therefore call to researchers for a secondary analysis of (ta)VNS datasets, which could offer more insight, particularly for studies assessing long-term effects and antidepressant response.

### Methodological considerations

A distinctive aspect of our study lies in our choice to stimulate the right outer ear. This decision was informed by rodent studies, that demonstrated a closer link between the right VN and limbic areas critical for reward behaviour and emotional processing. Rodent investigations have demonstrated that both right and left VNS impact cardiac reactivity, while effects were more often observed on the right side [54, 81, 82]. Because of the greater innervation of the sinus-atrial node by the right VN, invasive and most non-invasive taVNS applications have avoided this stimulation side fearing bradycardia. Nevertheless, there is currently no data suggesting safety concerns with transcutaneous VNS administered to the right side or bilaterally [83] and increases in HRV through VNS have been demonstrated for both right [61, 62, 84] and left VNS [60, 63, 85].

In addition, it was shown that specific stimulation parameters elicit an increase rather than a decrease of cytokines [86] indicating that a systematic investigation of stimulation parameters is necessary in future studies to further explore this dynamic in humans. Finally, in our study, we used post-hoc stratification with a median split for RMSSD as an index of cardiac vagal activation. Our cut-off value of 39.14 ms situates well within population-based reference data for the mean age of our sample (i.e., 50th percentile for age group 30–40: 37–41 ms albeit for 24-h recordings [87], and age group 35–39: 39.9–46 ms [88]). While continuous analyses yielded similar results, cut-off values (per age category) may be an attractive option for clinical application. To establish such cut-offs, future, population-specific studies controlling for confounding variables are needed.

## Limitations

While our study is highly novel, several limitations need to be mentioned. First, we used transcutaneous, not invasive VNS. We suggested the observed effects to be mediated through the activation of specific brain regions triggered by afferent fibre signaling. Although this signaling pathway lacks the precision of direct vagal fibre stimulation, recent rodent studies suggest it may achieve comparable outcomes [89]. We also did not measure respiration, but for some parameters, RMSSD is only reliably measured when the respiration rates are in the normal range (e.g., between 7.2–24 breaths/minute) [15], which could affect the results if breathing occurred outside of these ranges. Future studies should therefore measure breathing rates. In addition, while we conducted a power analysis for the primary aims of the study, the here reported analysis is conducted on secondary outcomes and may not be sufficiently powered to detect effects, particularly for the three-way-interaction. Therefore, a replication of results is needed. Finally, the effects we observed (notably for inflammation) are small and observed during acute stimulation. It remains to be seen whether taVNS or invasive VNS will also have this effect during longer stimulations and in clinical populations with higher levels of inflammation.

## Conclusion and outlook

In conclusion, our findings indicate that baseline RMSSD matters, at least for the biological outcomes of taVNS. A compromised CPA (here approximated by RMSSD) is associated with impaired cardiac and inflammatory responses to acute stress exposure and can be improved by acute taVNS. However, participants with high RMSSD may react with the opposite pattern, which could lead to null findings, as we observed in our analysis using diagnostic groups (e.g., MDD vs. controls). We call to all researchers who measured an ECG at baseline in studies using (ta)VNS to perform a secondary analysis. If our findings are replicated, this may help to find a stratification marker for successful taVNS for MDD, and other diagnoses.

## Supplementary information

Supplementary Tables

Supplementary Information

Supplementary Figure 1

## References

[1] Wang Z, Luo Y, Zhang Y, Chen L, Zou Y, Xiao J, et al. Heart rate variability in generalized anxiety disorder, major depressive disorder and panic disorder: a network meta-analysis and systematic review. J Affect Disord. 2023;330:259–66. 36914118 10.1016/j.jad.2023.03.018

[2] Pereira VH, Campos I, Sousa N. The role of autonomic nervous system in susceptibility and resilience to stress. Curr Opin Behav Sci. 2017;14:102–7.

[3] Larkin KT, Tiani AG, Brown LA Cardiac vagal tone and stress. 2021. 10.1093/acrefore/9780190264086.013.268.

[4] Rothe N, Steffen J, Penz M, Kirschbaum C, Walther A. Examination of peripheral basal and reactive cortisol levels in major depressive disorder and the burnout syndrome: a systematic review. Neurosci Biobehav Rev. 2020;114:232–70. 32088345 10.1016/j.neubiorev.2020.02.024

[5] Schiweck C, Piette D, Berckmans D, Claes S, Vrieze E. Heart rate and high frequency heart rate variability during stress as biomarker for clinical depression. a systematic review. Psychol Med. 2019;49:200–11. 30134999 10.1017/S0033291718001988

[6] Komegae EN, Farmer DGS, Brooks VL, McKinley MJ, McAllen RM, Martelli D. Vagal afferent activation suppresses systemic inflammation via the splanchnic anti-inflammatory pathway. Brain Behav Immun. 2018;73:441–9. 29883598 10.1016/j.bbi.2018.06.005 PMC6319822

[7] Chen X, Liang H, Hu K, Sun Q, Sun B, Bian L, et al. Vagus nerve stimulation suppresses corticotropin-releasing factor-induced adrenocorticotropic hormone release in rats. Neuroreport. 2021;32:792–6. https://journals.lww.com/neuroreport/fulltext/2021/06020/vagus_nerve_stimulation_suppresses.12.aspx. 10.1097/WNR.0000000000001656 33994530

[8] Borovikova LV, Ivanova S, Zhang M, Yang H, Botchkina GI, Watkins LR, et al. Vagus nerve stimulation attenuates the systemic inflammatory response to endotoxin. Nature. 2000;405:458–62. 10839541 10.1038/35013070

[9] Liu J, Fang J, Wang Z, Rong P, Hong Y, Fan Y, et al. Transcutaneous vagus nerve stimulation modulates amygdala functional connectivity in patients with depression. J Affect Disord. 2016;205:319–26. 27559632 10.1016/j.jad.2016.08.003

[10] Laule C, Sayar-Atasoy N, Aklan I, Kim H, Ates T, Davis D, et al. Stress integration by an ascending adrenergic-melanocortin circuit. Neuropsychopharmacology. 2024;49:1361–72. 10.1038/s41386-024-01810-9. 38326456 10.1038/s41386-024-01810-9 PMC11251172

[11] Zhang S-Q, Xia Z-X, Deng Q, Yang P-F, Long L-H, Wang F, et al. Repeated vagus nerve stimulation produces anxiolytic effects via upregulation of AMPAR function in centrolateral amygdala of male rats. Neurobiol Stress. 2022;18:100453. 35685681 10.1016/j.ynstr.2022.100453 PMC9170826

[12] Alvarez-Dieppa AC, Griffin K, Cavalier S, McIntyre CK. Vagus nerve stimulation enhances extinction of conditioned fear in rats and modulates Arc protein, CaMKII, and GluN2B-containing NMDA receptors in the basolateral amygdala. Neural Plast. 2016;2016:4273280. 27957346 10.1155/2016/4273280 PMC5120198

[13] Shin HC, Jo BG, Lee C-Y, Lee K-W, Namgung U. Hippocampal activation of 5-HT1B receptors and BDNF production by vagus nerve stimulation in rats under chronic restraint stress. Eur J Neurosci. 2019;50:1820–30. 30735600 10.1111/ejn.14368

[14] Lyubashina O, Panteleev S. Effects of cervical vagus nerve stimulation on amygdala-evoked responses of the medial prefrontal cortex neurons in rat. Neurosci Res. 2009;65:122–5. 19523995 10.1016/j.neures.2009.06.002

[15] Quigley KS, Gianaros PJ, Norman GJ, Jennings JR, Berntson GG, de Geus EJC. Publication guidelines for human heart rate and heart rate variability studies in psychophysiology—Part 1: physiological underpinnings and foundations of measurement. Psychophysiology. 2024;61:e14604. 38873876 10.1111/psyp.14604 PMC11539922

[16] Shaffer F, Ginsberg JP. An overview of heart rate variability metrics and norms. Front Public Health. 2017;5:258. https://www.frontiersin.org/journals/public-health/articles/10.3389/fpubh.2017.00258. 10.3389/fpubh.2017.00258 PMC5624990 29034226

[17] Borgmann D, Rigoux L, Kuzmanovic B, Thanarajah SE, Münte TF, Fenselau H, et al. Modulation of fMRI brainstem responses by transcutaneous vagus nerve stimulation. Neuroimage. 2021;244:118566. 34509623 10.1016/j.neuroimage.2021.118566

[18] Teckentrup V, Krylova M, Jamalabadi H, Neubert S, Neuser MP, Hartig R, et al. Brain signaling dynamics after vagus nerve stimulation. Neuroimage. 2021;245:118679.

[19] Frangos E, Ellrich J, Komisaruk BR. Non-invasive access to the vagus nerve central projections via electrical stimulation of the external ear: fMRI evidence in humans. Brain Stimul. 2015;8:624–36. 25573069 10.1016/j.brs.2014.11.018 PMC4458242

[20] Oehrn CR, Molitor L, Krause K, Niehaus H, Schmidt L, Hakel L, et al. Non-invasive vagus nerve stimulation in epilepsy patients enhances cooperative behavior in the prisoner’s dilemma task. Sci Rep. 2022;12:10255. 35715460 10.1038/s41598-022-14237-3 PMC9205877

[21] Kühnel A, Teckentrup V, Neuser MP, Huys QJM, Burrasch C, Walter M, et al. Stimulation of the vagus nerve reduces learning in a go/no-go reinforcement learning task. Eur Neuropsychopharmacol. 2020;35:17–29. 32404279 10.1016/j.euroneuro.2020.03.023

[22] Neuser MP, Teckentrup V, Kühnel A, Hallschmid M, Walter M, Kroemer NB. Vagus nerve stimulation boosts the drive to work for rewards. Nat Commun. 2020;11:3555. 32678082 10.1038/s41467-020-17344-9 PMC7366927

[23] Schiweck C, Sausmekat S, Zhao T, Jacobsen L, Reif A, Thanarajah SE No consistent evidence for the anti-inflammatory effect of vagus nerve stimulation in humans: a systematic review and meta-analysis. Brain Behav Immun 2024;116:237–258. 10.1016/j.bbi.2023.12.008 38070618

[24] Ferstl M, Teckentrup V, Lin WM, Kräutlein F, Kühnel A, Klaus J, et al. Non-invasive vagus nerve stimulation boosts mood recovery after effort exertion. Psychol Med. 2022;52:3029–39. 33586647 10.1017/S0033291720005073 PMC9693679

[25] Bassi GS, Kanashiro A, Coimbra NC, Terrando N, Maixner W, Ulloa L. Anatomical and clinical implications of vagal modulation of the spleen. Neurosci Biobehav Rev. 2020;112:363–73. 32061636 10.1016/j.neubiorev.2020.02.011 PMC7211143

[26] Cuberos Paredes E, Goyes D, Mak S, Yardimian R, Ortiz N, McLaren A, et al. Transcutaneous auricular vagus nerve stimulation inhibits mental stress-induced cortisol release—potential implications for inflammatory conditions. Physiol Rep. 2025;13:e70251. 39936474 10.14814/phy2.70251 PMC11815478

[27] De Smet S, Ottaviani C, Verkuil B, Kappen M, Baeken C, Vanderhasselt M-A. Effects of non-invasive vagus nerve stimulation on cognitive and autonomic correlates of perseverative cognition. Psychophysiology. 2023;60:e14250. 36683127 10.1111/psyp.14250

[28] Tan C, Qiao M, Ma Y, Luo Y, Fang J, Yang Y. The efficacy and safety of transcutaneous auricular vagus nerve stimulation in the treatment of depressive disorder: a systematic review and meta-analysis of randomized controlled trials. J Affect Disord. 2023;337:37–49. 37230264 10.1016/j.jad.2023.05.048

[29] Sgoifo A, Carnevali L, Alfonso Mde L, Amore M. Autonomic dysfunction and heart rate variability in depression. Stress. 2015;18:343–52. 26004818 10.3109/10253890.2015.1045868

[30] Han W, Tellez LA, Perkins MH, Perez IO, Qu T, Ferreira J, et al. A neural circuit for gut-induced reward. Cell. 2018;175:665–.e23. 30245012 10.1016/j.cell.2018.08.049 PMC6195474

[31] Brougher J, Aziz U, Adari N, Chaturvedi M, Jules A, Shah I, et al. Self-administration of right vagus nerve stimulation activates midbrain dopaminergic nuclei. Front Neurosci. 2021;15:782786. https://www.frontiersin.org/journals/neuroscience/articles/10.3389/fnins.2021.782786. 10.3389/fnins.2021.782786 PMC8716493 34975384

[32] Tarvainen MP, Niskanen J-P, Lipponen JA, Ranta-Aho PO, Karjalainen PA. Kubios HRV–heart rate variability analysis software. Comput Methods Prog Biomed. 2014;113:210–20. 10.1016/j.cmpb.2013.07.024 24054542

[33] Olshansky B, Ricci F, Fedorowski A Importance of resting heart rate. Trends Cardiovasc Med 2022. 10.1016/j.tcm.2022.05.006 35623552

[34] Mayer M. missRanger: Fast Imputation of Missing Values. R package version 2.6.2 2019. https://mayer79.github.io/missRanger/, https://github.com/mayer79/missRanger.

[35] Electrophysiology TF of the ES of C the NAS of P.. Heart rate variability: standards of measurement, physiological interpretation, and clinical use. Circulation. 1996;93:1043–65. 8598068

[36] Emerson SR, Kurti SP, Harms CA, Haub MD, Melgarejo T, Logan C, et al. Magnitude and timing of the postprandial inflammatory response to a high-fat meal in healthy adults: a systematic review. Adv Nutr. 2017;8:213–25. 28298267 10.3945/an.116.014431 PMC5347112

[37] Blackburn P, Després J-P, Lamarche B, Tremblay A, Bergeron J, Lemieux I, et al. Postprandial variations of plasma inflammatory markers in abdominally obese men. Obesity. 2006;14:1747–54. 17062804 10.1038/oby.2006.201

[38] Sheehan DV, Lecrubier Y, Sheehan KH, Amorim P, Janavs J, Weiller E, et al. The mini-international neuropsychiatric interview (MINI): the development and validation of a structured diagnostic psychiatric interview for DSM-IV and ICD-10. J Clin psychiatry. 1998;59:22–33. 9881538

[39] Davidson J, Turnbull CD, Strickland R, Miller R, Graves K. The montgomery-åsberg depression scale: reliability and validity. Acta Psychiatr Scand. 1986;73:544–8. 3751660 10.1111/j.1600-0447.1986.tb02723.x

[40] Cohen S, Kamarck T, Mermelstein R. Perceived stress scale. Measuring Stress: A guide health Soc scientists. 1994;10:1–2.

[41] Beck AT, Steer RA, Brown GK Beck depression inventory. Harcourt Brace Jovanovich New York:, 1987.

[42] Bernstein DP, Fink L, Handelsman L, Foote J Childhood trauma questionnaire. Assessment of family violence: A handbook for researchers and practitioners 1998.

[43] Dedovic K, Renwick R, Mahani NK, Engert V, Lupien SJ, Pruessner JC. The montreal imaging stress task: using functional imaging to investigate the effects of perceiving and processing psychosocial stress in the human brain. J Psychiatry Neurosci. 2005;30:319–25. 16151536 PMC1197276

[44] Douglas Bates MM, Bolker B, Walker S. Fitting linear mixed-effects models using lme4. J Stat Softw. 2015;67:1–48.

[45] Kuznetsova A, Brockhoff PB, Christensen RHB. lmerTest package: tests in linear mixed effects models. J Stat Softw 2017;82:1–26. 10.18637/jss.v082.i13.

[46] Lenth R, Lenth MR. Package ‘lsmeans’. Am Stat. 2018;34:216–21.

[47] R Core Team. R: A Language and Environment for Statistical Computing_. 2023.

[48] Wu Q, Miao X, Cao Y, Chi A, Xiao T. Heart rate variability status at rest in adult depressed patients: a systematic review and meta-analysis. Front Public Health. 2023;11:1243213. https://www.frontiersin.org/journals/public-health/articles/10.3389/fpubh.2023.1243213. 10.3389/fpubh.2023.1243213 PMC10760642 38169979

[49] Sin NL, Sloan RP, McKinley PS, Almeida DM. Linking daily stress processes and laboratory-based heart rate variability in a national sample of midlife and older adults. Psychosom Med. 2016;78:573. 26867082 10.1097/PSY.0000000000000306 PMC4891238

[50] Wesarg C, Van den Akker AL, Oei NYL, Wiers RW, Staaks J, Thayer JF, et al. Childhood adversity and vagal regulation: A systematic review and meta-analysis. Neurosci Biobehav Rev. 2022;143:104920. 10.1016/j.neubiorev.2022.104920. 10.1016/j.neubiorev.2022.104920 36272580

[51] Okonogi T, Kuga N, Yamakawa M, Kayama T, Ikegaya Y, Sasaki T. Stress-induced vagal activity influences anxiety-relevant prefrontal and amygdala neuronal oscillations in male mice. Nat Commun. 2024;15:183. 38195621 10.1038/s41467-023-44205-y PMC10776769

[52] Weber CS, Thayer JF, Rudat M, Wirtz PH, Zimmermann-Viehoff F, Thomas A, et al. Low vagal tone is associated with impaired post stress recovery of cardiovascular, endocrine, and immune markers. Eur J Appl Physiol. 2010;109:201–11. 20052593 10.1007/s00421-009-1341-x

[53] Souza GGL, Mendonça-de-Souza ACF, Barros EM, Coutinho EFS, Oliveira L, Mendlowicz MV, et al. Resilience and vagal tone predict cardiac recovery from acute social stress. Stress. 2007;10:368–74. 17853065 10.1080/10253890701419886

[54] Lee SW, Anderson A, Guzman PA, Nakano A, Tolkacheva EG, Wickman K. Atrial GIRK channels mediate the effects of vagus nerve stimulation on heart rate dynamics and arrhythmogenesis. Front Physiol. 2018;9:943. 30072916 10.3389/fphys.2018.00943 PMC6060443

[55] Dhein S, Van Koppen CJ, Brodde O-E. Muscarinic receptors in the mammalian heart. Pharmacol Res. 2001;44:161–82. 11529684 10.1006/phrs.2001.0835

[56] Ardell JL, Rajendran PS, Nier HA, KenKnight BH, Armour JA. Central-peripheral neural network interactions evoked by vagus nerve stimulation: functional consequences on control of cardiac function. Am J Physiol -Heart Circulatory Physiol. 2015;309:H1740–H1752. 10.1152/ajpheart.00557.2015 PMC4666982 26371171

[57] Weise D, Adamidis M, Pizzolato F, Rumpf J-J, Fricke C, Classen J. Assessment of brainstem function with auricular branch of vagus nerve stimulation in parkinson’s disease. PLoS One. 2015;10:e0120786. 25849807 10.1371/journal.pone.0120786 PMC4388709

[58] Altınkaya Z, Öztürk L, Büyükgüdük İ, Yanık H, Yılmaz DD, Yar B, et al. Non-invasive vagus nerve stimulation in a hungry state decreases heart rate variability. Physiol Behav. 2023;258:114016. 36334796 10.1016/j.physbeh.2022.114016

[59] Kaduk K, Petrella A, Müller SJ, Koenig J, Kroemer NB. Non-Invasive Auricular Vagus Nerve Stimulation Decreases Heart Rate Variability Independent of Caloric Load. Psychophysiology. 2025;62:e70017. 10.1111/psyp.70017. 10.1111/psyp.70017 PMC11862327 40007175

[60] Antonino D, Teixeira AL, Maia-Lopes PM, Souza MC, Sabino-Carvalho JL, Murray AR, et al. Non-invasive vagus nerve stimulation acutely improves spontaneous cardiac baroreflex sensitivity in healthy young men: a randomized placebo-controlled trial. Brain Stimul. 2017;10:875–81. 28566194 10.1016/j.brs.2017.05.006

[61] Machetanz K, Berelidze L, Guggenberger R, Gharabaghi A. Brain–heart interaction during transcutaneous auricular vagus nerve stimulation. Front Neurosci. 2021;15:632697. 33790736 10.3389/fnins.2021.632697 PMC8005577

[62] Gauthey A, Morra S, Van de Borne P, Deriaz D, Maes N, Le Polain De Waroux J-B. Sympathetic effect of auricular transcutaneous vagus nerve stimulation on healthy subjects: a crossover controlled clinical trial comparing vagally mediated and active control stimulation using microneurography. Front Physiol. 2020;11:599896. 33343394 10.3389/fphys.2020.599896 PMC7744823

[63] Geng D, Yang K, Fu Z, Zhang Y, Wang C, An H. Circadian stage-dependent and stimulation duration effects of transcutaneous auricular vagus nerve stimulation on heart rate variability. PLoS One. 2022;17:e0277090. 36327249 10.1371/journal.pone.0277090 PMC9632923

[64] Jansen K, Vandeput S, Milosevic M, Ceulemans B, Van Huffel S, Brown L, et al. Autonomic effects of refractory epilepsy on heart rate variability in children: influence of intermittent vagus nerve stimulation. Dev Med Child Neurol. 2011;53:1143–9. 21883174 10.1111/j.1469-8749.2011.04103.x

[65] Sinkovec M, Trobec R, Meglic B. Cardiovascular responses to low-level transcutaneous vagus nerve stimulation. Autonomic Neurosci. 2021;236:102851. 10.1016/j.autneu.2021.102851 34274638

[66] Galli R, Limbruno U, Pizzanelli C, Giorgi FS, Lutzemberger L, Strata G, et al. Analysis of RR variability in drug-resistant epilepsy patients chronically treated with vagus nerve stimulation. Autonomic Neurosci. 2003;107:52–59. 10.1016/s1566-0702(03)00081-x 12927227

[67] Borges U, Laborde S, Raab M. Influence of transcutaneous vagus nerve stimulation on cardiac vagal activity: not different from sham stimulation and no effect of stimulation intensity. PLoS One. 2019;14:e0223848. 31603939 10.1371/journal.pone.0223848 PMC6788680

[68] Wolf V, Kühnel A, Teckentrup V, Koenig J, Kroemer NB. Does transcutaneous auricular vagus nerve stimulation affect vagally mediated heart rate variability? a living and interactive bayesian meta-analysis. Psychophysiology. 2021;58:e13933. 34473846 10.1111/psyp.13933

[69] van den Berg ME, Rijnbeek PR, Niemeijer MN, Hofman A, van Herpen G, Bots ML, et al. Normal values of corrected heart-rate variability in 10-second electrocardiograms for all ages. Front Physiol. 2018;9:424. https://www.frontiersin.org/journals/physiology/articles/10.3389/fphys.2018.00424. 10.3389/fphys.2018.00424 PMC5934689 29755366

[70] Hein E, Nowak M, Kiess O, Biermann T, Bayerlein K, Kornhuber J, et al. Auricular transcutaneous electrical nerve stimulation in depressed patients: a randomized controlled pilot study. J Neural Transm. 2013;120:821–7. 23117749 10.1007/s00702-012-0908-6

[71] Pellissier S, Dantzer C, Mondillon L, Trocme C, Gauchez A-S, Ducros V, et al. Relationship between vagal tone, cortisol, TNF-alpha, epinephrine and negative affects in crohn’s disease and irritable bowel syndrome. PLoS One. 2014;9:e105328. 25207649 10.1371/journal.pone.0105328 PMC4160179

[72] Marsland AL, Gianaros PJ, Prather AA, Jennings JR, Neumann SA, Manuck SB. Stimulated production of proinflammatory cytokines covaries inversely with heart rate variability. Psychosom Med. 2007;69:709–16. 17942840 10.1097/PSY.0b013e3181576118

[73] Veiz E, Kieslich S-K, Czesnik D, Herrmann-Lingen C, Meyer T, Staab J. Increased concentrations of circulating interleukins following non-invasive vagus nerve stimulation: results from a randomized, sham-controlled, crossover study in healthy subjects. Neuroimmunomodulation. 2022;29:450–9. 35576915 10.1159/000524646

[74] Corcoran C, Connor TJ, O’Keane V, Garland MR. The effects of vagus nerve stimulation on pro-and anti-inflammatory cytokines in humans: a preliminary report. Neuroimmunomodulation. 2005;12:307–9. 16166810 10.1159/000087109

[75] Paciorek A, Skora L. Vagus nerve stimulation as a gateway to interoception. Front Psychol. 2020;11:1659. https://www.frontiersin.org/journals/psychology/articles/10.3389/fpsyg.2020.01659. 10.3389/fpsyg.2020.01659 PMC7403209 32849014

[76] Cho CH, Hung KM, Ogle CW. The aetiology of gastric ulceration induced by electrical vagal stimulation in rats. Eur J Pharmacol. 1985;110:211–7. 2985410 10.1016/0014-2999(85)90213-4

[77] Bright RA, Dowdy K, Rankin SK, Blok SV, Palmer LA, Bright-Ponte SJ New and increasing rates of adverse events can be found in unstructured text in electronic health records using the shakespeare method. medRxiv [Preprint]. 2021 Available from: https://www.medrxiv.org/content/10.1101/2021.01.12.21249674v2.full.pdf. 10.2196/27017 PMC10414364 37725533

[78] Kollai M, Bonyhay I, Jokkel G, Szonyi L. Cardiac vagal hyperactivity in adolescent anorexia nervosa. Eur Heart J. 1994;15:1113–8. 7988604 10.1093/oxfordjournals.eurheartj.a060636

[79] Bonaz B, Sinniger V, Hoffmann D, Clarençon D, Mathieu N, Dantzer C, et al. Chronic vagus nerve stimulation in Crohn’s disease: a 6-month follow-up pilot study. Neurogastroenterology Motil. 2016;28:948–53. 10.1111/nmo.12792 26920654

[80] Kavakbasi E, Kraus C, Reif-Leonhard C, Blackwell J-M, Dibué M, Treiber M, et al. Titration of vagus nerve stimulation for difficult-to-treat depression and onset of response: early insights from the RESTORE-LIFE study. J Affect Disord. 2025;378:39–46. 40021060 10.1016/j.jad.2025.02.047

[81] Huang J, Wang Y, Jiang D, Zhou J, Huang X. The sympathetic-vagal balance against endotoxemia. J Neural Transm. 2010;117:729–35. 20458507 10.1007/s00702-010-0407-6

[82] Yaghouby F, Jang K, Hoang U, Asgari S, Vasudevan S. Sex differences in vagus nerve stimulation effects on rat cardiovascular and immune systems. Front Neurosci. 2020;14:560668. 33240036 10.3389/fnins.2020.560668 PMC7677457

[83] Kim AY, Marduy A, de Melo PS, Gianlorenco AC, Kim CK, Choi H, et al. Safety of transcutaneous auricular vagus nerve stimulation (taVNS): A systematic review and meta-analysis. Sci Rep. 2022;12:22055. 36543841 10.1038/s41598-022-25864-1 PMC9772204

[84] De Couck M, Cserjesi R, Caers R, Zijlstra WP, Widjaja D, Wolf N, et al. Effects of short and prolonged transcutaneous vagus nerve stimulation on heart rate variability in healthy subjects. Autonomic Neurosci. 2017;203:88–96. 10.1016/j.autneu.2016.11.003 28017263

[85] Forte G, Favieri F, Leemhuis E, De Martino ML, Giannini AM, De Gennaro L, et al. Ear your heart: transcutaneous auricular vagus nerve stimulation on heart rate variability in healthy young participants. PeerJ. 2022;10:e14447. 36438582 10.7717/peerj.14447 PMC9686410

[86] Tsaava T, Datta-Chaudhuri T, Addorisio ME, Masi EB, Silverman HA, Newman JE, et al. Specific vagus nerve stimulation parameters alter serum cytokine levels in the absence of inflammation. Bioelectron Med. 2020;6:1–10. 32309522 10.1186/s42234-020-00042-8 PMC7146955

[87] Bauer A, Camm AJ, Cerutti S, Guzik P, Huikuri H, Lombardi F, et al. Reference values of heart rate variability. Heart Rhythm. 2017;14:302–3. 27986557 10.1016/j.hrthm.2016.12.015

[88] Tegegne BS, Man T, van Roon AM, Snieder H, Riese H. Reference values of heart rate variability from 10 s resting electrocardiograms: the Lifelines Cohort Study. Eur J Prev Cardiol. 2020;27:2191–4. 31500461 10.1177/2047487319872567 PMC7734556

[89] Owens MM, Jacquemet V, Napadow V, Lewis N, Beaumont E. Brainstem neuronal responses to transcutaneous auricular and cervical vagus nerve stimulation in rats. J Physiol. 2024;602:4027–52. 39031516 10.1113/JP286680 PMC11326965

