# Supplementary material for: The heart knows best: baseline heart rate variability as guide to transcutaneous auricular vagus nerve stimulation in depression
Source: Transl Psychiatry. 2025 Dec 6;15:521. doi: 10.1038/s41398-025-03780-y (PMC12689627; doi:10.1038/s41398-025-03780-y)
Supplement: Supplementary file 1 — Supplementary Tables [file 41398_2025_3780_MOESM1_ESM.docx]

**SUPPLEMENTARY TABLES**

**Supplementary Table 1. Supplementary Analyses with Diagnosis MDD/HC as moderating factor.** Emotions were transformed using tukey transformatios, all other variables were transformed on the log scale. The PR(>F) value indicated the p value of all participants, the Significance without TCA indicates p values when those taking TCA were omitted.

| **Supplementary Table 1A. Emotions** | | | | | | | | | | | | | | |
| --- | --- | --- | --- | --- | --- | --- | --- | --- | --- | --- | --- | --- | --- | --- |
| **Stress** | **Sum Sq** | | | **Mean Sq** | | **NumDF** | | **DenDF** | | **F** | | **Pr(>F)** | | **Significance without TCA** |
| Intervention | 113.000 | | | 113.200 | | 1.000 | | 509.710 | | 0.621 | | 0.431 | | 0.116 |
| Group | 8315.000 | | | 8315.100 | | 1.000 | | 100.710 | | 45.641 | | **0.000** | | **0.000** |
| Timepoint | 46701.000 | | | 23350.300 | | 2.000 | | 493.430 | | 128.167 | | **0.000** | | **0.000** |
| Sex | 65.000 | | | 64.800 | | 1.000 | | 99.540 | | 0.356 | | 0.552 | | 0.794 |
| Age | 319.000 | | | 318.900 | | 1.000 | | 100.050 | | 1.750 | | 0.189 | | 0.193 |
| Testday | 476.000 | | | 475.900 | | 1.000 | | 517.750 | | 2.612 | | 0.107 | | 0.460 |
| Stimulation Intensity | 53.000 | | | 52.900 | | 1.000 | | 590.820 | | 0.291 | | 0.590 | | 0.218 |
| Intervention:Group | 229.000 | | | 228.800 | | 1.000 | | 504.360 | | 1.256 | | 0.263 | | 0.673 |
| Interverntion:Timepoint | 316.000 | | | 157.900 | | 2.000 | | 493.430 | | 0.867 | | 0.421 | | 0.394 |
| Group:Timepoint | 4067.000 | | | 2033.700 | | 2.000 | | 493.430 | | 11.163 | | **0.000** | | **0.000** |
| Intervention:Group:Timepoint | 132.000 | | | 66.200 | | 2.000 | | 493.430 | | 0.363 | | 0.696 | | 0.644 |
|  | | | | | | | | | | | | | | |
| **Negative Emotions** | **Sum Sq** | | | **Mean Sq** | | **NumDF** | | **DenDF** | | **F** | | **Pr(>F)** | | **Significance without TCA** |
| Intervention | 54.200 | | | 54.200 | | 1.000 | | 504.950 | | 1.745 | | 0.187 | | 0.591 |
| Group | 2252.700 | | | 2252.700 | | 1.000 | | 101.830 | | 72.534 | | **0.000** | | **0.000** |
| Timepoint | 6612.000 | | | 3306.000 | | 2.000 | | 494.550 | | 106.447 | | **0.000** | | **0.000** |
| Sex | 86.700 | | | 86.700 | | 1.000 | | 101.060 | | 2.790 | | 0.098 | | 0.158 |
| Age | 75.000 | | | 75.000 | | 1.000 | | 101.400 | | 2.415 | | 0.123 | | 0.170 |
| Testday | 90.300 | | | 90.300 | | 1.000 | | 510.600 | | 2.908 | | 0.089 | | 0.381 |
| Stimulation Intensity | 0.600 | | | 0.600 | | 1.000 | | 589.390 | | 0.020 | | 0.888 | | 0.391 |
| Intervention:Group | 2.700 | | | 2.700 | | 1.000 | | 501.430 | | 0.087 | | 0.768 | | 0.718 |
| Intervention:Timepoint | 97.500 | | | 48.800 | | 2.000 | | 494.550 | | 1.570 | | 0.209 | | 0.219 |
| Group:Timepoint | 606.500 | | | 303.300 | | 2.000 | | 494.550 | | 9.764 | | **0.000** | | **0.000** |
| Intervention:Group:Timepoint | 7.900 | | | 4.000 | | 2.000 | | 494.550 | | 0.128 | | 0.880 | | 0.870 |
|  | | | | | | | | | | | | | | |
| **Positive Emotions** | **Sum Sq** | | | **Mean Sq** | | **NumDF** | | **DenDF** | | **F** | | **Pr(>F)** | | **Significance without TCA** |
| Intervention | 2952.000 | | | 2952.000 | | 1.000 | | 505.440 | | 1.178 | | 0.278 | | 0.837 |
| Group | 281939.000 | | | 281939.000 | | 1.000 | | 103.480 | | 112.511 | | **0.000** | | **0.000** |
| Timepoint | 51329.000 | | | 25664.000 | | 2.000 | | 496.200 | | 10.242 | | **0.000** | | **0.000** |
| Sex | 1428.000 | | | 1428.000 | | 1.000 | | 102.780 | | 0.570 | | 0.452 | | 0.624 |
| Age | 2569.000 | | | 2569.000 | | 1.000 | | 103.090 | | 1.025 | | 0.314 | | 0.399 |
| Testday | 31677.000 | | | 31677.000 | | 1.000 | | 510.550 | | 12.641 | | **0.000** | | **0.010** |
| Stimulation Intensity | 3758.000 | | | 3758.000 | | 1.000 | | 584.140 | | 1.500 | | 0.221 | | 0.691 |
| Intervention:Group | 848.000 | | | 848.000 | | 1.000 | | 502.310 | | 0.339 | | 0.561 | | 0.871 |
| Interverntion:Timepoint | 819.000 | | | 409.000 | | 2.000 | | 496.200 | | 0.163 | | 0.849 | | 0.878 |
| Group:Timepoint | 57.000 | | | 29.000 | | 2.000 | | 496.200 | | 0.011 | | 0.989 | | 0.953 |
| Intervention:Group:Timepoint | 10039.000 | | | 5019.000 | | 2.000 | | 496.200 | | 2.003 | | 0.136 | | 0.079 |
| **Supplementary Table 1B. Heart Rate Variability** | | | | | | | | | | | | | | |
| **HR** | | **Sum Sq** | **Mean Sq** | | **NumDF** | | **DenDF** | | **F** | | **Pr(>F)** | | **Significance without TCA** | |
| Intervention | | 0.001 | 0.001 | | 1.000 | | 455.300 | | 0.355 | | 0.552 | | 0.478 | |
| Group | | 0.004 | 0.004 | | 1.000 | | 101.610 | | 1.027 | | 0.313 | | 0.434 | |
| Timepoint | | 0.434 | 0.217 | | 2.000 | | 444.250 | | 57.207 | | **0.000** | | **0.000** | |
| Sex | | 0.044 | 0.044 | | 1.000 | | 101.280 | | 11.586 | | **0.001** | | **0.000** | |
| Age | | 0.034 | 0.034 | | 1.000 | | 101.820 | | 8.857 | | **0.004** | | **0.002** | |
| Testday | | 0.000 | 0.000 | | 1.000 | | 460.580 | | 0.123 | | 0.726 | | 0.621 | |
| Stimulation Intensity | | 0.014 | 0.014 | | 1.000 | | 512.020 | | 3.712 | | 0.055 | | **0.039** | |
| Intervention:Group | | 0.002 | 0.002 | | 1.000 | | 453.410 | | 0.455 | | 0.501 | | 0.440 | |
| Interverntion:Timepoint | | 0.004 | 0.002 | | 2.000 | | 444.150 | | 0.466 | | 0.628 | | 0.654 | |
| Group:Timepoint | | 0.008 | 0.004 | | 2.000 | | 444.240 | | 1.080 | | 0.340 | | 0.439 | |
| Intervention:Group:Timepoint | | 0.003 | 0.002 | | 2.000 | | 444.150 | | 0.419 | | 0.658 | | 0.663 | |
|  | |  |  | |  | |  | |  | |  | |  | |
| **RMSSD** | | **Sum Sq** | **Mean Sq** | | **NumDF** | | **DenDF** | | **F** | | **Pr(>F)** | | **Significance without TCA** | |
| Intervention | | 0.119 | 0.119 | | 1.000 | | 455.740 | | 1.746 | | 0.187 | | 0.358 | |
| Group | | 0.421 | 0.421 | | 1.000 | | 101.170 | | 6.179 | | **0.015** | | **0.027** | |
| Timepoint | | 3.584 | 1.792 | | 2.000 | | 443.880 | | 26.282 | | **0.000** | | **0.000** | |
| Sex | | 0.087 | 0.087 | | 1.000 | | 100.820 | | 1.269 | | 0.263 | | 0.559 | |
| Age | | 0.076 | 0.076 | | 1.000 | | 101.400 | | 1.112 | | 0.294 | | 0.493 | |
| Testday | | 0.008 | 0.008 | | 1.000 | | 461.350 | | 0.113 | | 0.737 | | 0.923 | |
| Stimulation Intensity | | 0.001 | 0.001 | | 1.000 | | 515.740 | | 0.019 | | 0.890 | | 0.801 | |
| Intervention:Group | | 0.004 | 0.004 | | 1.000 | | 453.710 | | 0.058 | | 0.809 | | 0.918 | |
| Interverntion:Timepoint | | 0.257 | 0.128 | | 2.000 | | 443.770 | | 1.884 | | 0.153 | | 0.185 | |
| Group:Timepoint | | 0.362 | 0.181 | | 2.000 | | 443.870 | | 2.652 | | 0.072 | | 0.094 | |
| Intervention:Group:Timepoint | | 0.014 | 0.007 | | 2.000 | | 443.770 | | 0.104 | | 0.901 | | 0.929 | |
|  | |  |  | |  | |  | |  | |  | |  | |
| **HF-HRV** | | **Sum Sq** | **Mean Sq** | | **NumDF** | | **DenDF** | | **F** | | **Pr(>F)** | | **Significance without TCA** | |
| Intervention | | 0.214 | 0.214 | | 1.000 | | 460.280 | | 0.587 | | 0.444 | | 0.617 | |
| Group | | 2.597 | 2.597 | | 1.000 | | 101.440 | | 7.126 | | **0.009** | | **0.017** | |
| Timepoint | | 10.456 | 5.228 | | 2.000 | | 444.480 | | 14.347 | | **0.000** | | **0.000** | |
| Sex | | 1.799 | 1.799 | | 1.000 | | 100.980 | | 4.936 | | **0.029** | | 0.090 | |
| Age | | 1.628 | 1.628 | | 1.000 | | 101.760 | | 4.469 | | **0.037** | | 0.073 | |
| Testday | | 0.582 | 0.582 | | 1.000 | | 467.360 | | 1.597 | | 0.207 | | 0.352 | |
| Stimulation Intensity | | 0.083 | 0.083 | | 1.000 | | 532.500 | | 0.228 | | 0.633 | | 0.841 | |
| Intervention:Group | | 0.019 | 0.019 | | 1.000 | | 457.590 | | 0.053 | | 0.819 | | 0.997 | |
| Interverntion:Timepoint | | 0.254 | 0.127 | | 2.000 | | 444.320 | | 0.348 | | 0.706 | | 0.782 | |
| Group:Timepoint | | 1.430 | 0.715 | | 2.000 | | 444.460 | | 1.963 | | 0.142 | | 0.198 | |
| Intervention:Group:Timepoint | | 0.460 | 0.230 | | 2.000 | | 444.330 | | 0.631 | | 0.533 | | 0.578 | |
|  | |  |  | |  | |  | |  | |  | |  | |
| **LF-HRV** | | **Sum Sq** | **Mean Sq** | | **NumDF** | | **DenDF** | | **F** | | **Pr(>F)** | | **Significance without TCA** | |
| Intervention | | 0.535 | 0.535 | | 1.000 | | 468.800 | | 1.277 | | 0.259 | | 0.381 | |
| Group | | 1.908 | 1.908 | | 1.000 | | 100.770 | | 4.557 | | **0.035** | | **0.048** | |
| Timepoint | | 19.977 | 9.989 | | 2.000 | | 444.740 | | 23.856 | | **0.000** | | **0.000** | |
| Sex | | 0.121 | 0.121 | | 1.000 | | 100.120 | | 0.289 | | 0.592 | | 0.230 | |
| Age | | 5.028 | 5.028 | | 1.000 | | 101.310 | | 12.009 | | **0.001** | | **0.001** | |
| Testday | | 0.224 | 0.224 | | 1.000 | | 478.270 | | 0.534 | | 0.465 | | 0.668 | |
| Stimulation Intensity | | 0.175 | 0.175 | | 1.000 | | 542.990 | | 0.418 | | 0.518 | | 0.722 | |
| Intervention:Group | | 0.601 | 0.601 | | 1.000 | | 464.760 | | 1.436 | | 0.231 | | 0.167 | |
| Interverntion:Timepoint | | 2.743 | 1.372 | | 2.000 | | 444.480 | | 3.276 | | **0.039** | | **0.044** | |
| Group:Timepoint | | 0.607 | 0.304 | | 2.000 | | 444.710 | | 0.725 | | 0.485 | | 0.391 | |
| Intervention:Group:Timepoint | | 0.763 | 0.382 | | 2.000 | | 444.480 | | 0.912 | | 0.403 | | 0.421 | |
|  | |  |  | |  | |  | |  | |  | |  | |
| **LF/HF ratio** | | **Sum Sq** | **Mean Sq** | | **NumDF** | | **DenDF** | | **F** | | **Pr(>F)** | |  | |
| Intervention | | 0.082 | 0.082 | | 1.000 | | 469.840 | | 0.194 | | 0.660 | | 0.701 | |
| Group | | 0.740 | 0.740 | | 1.000 | | 95.360 | | 1.746 | | 0.190 | | 0.291 | |
| Timepoint | | 1.773 | 0.887 | | 2.000 | | 439.880 | | 2.093 | | 0.125 | | 0.213 | |
| Sex | | 5.732 | 5.732 | | 1.000 | | 94.650 | | 13.532 | | 0.000 | | **0.001** | |
| Age | | 0.422 | 0.422 | | 1.000 | | 96.020 | | 0.996 | | 0.321 | | 0.173 | |
| Testday | | 0.117 | 0.117 | | 1.000 | | 480.690 | | 0.277 | | 0.599 | | 0.589 | |
| Stimulation Intensity | | 0.021 | 0.021 | | 1.000 | | 529.680 | | 0.050 | | 0.823 | | 0.844 | |
| Intervention:Group | | 0.839 | 0.839 | | 1.000 | | 464.840 | | 1.980 | | 0.160 | | 0.155 | |
| Interverntion:Timepoint | | 1.334 | 0.667 | | 2.000 | | 439.530 | | 1.575 | | 0.208 | | 0.182 | |
| Group:Timepoint | | 0.263 | 0.131 | | 2.000 | | 439.850 | | 0.310 | | 0.733 | | 0.879 | |
| Intervention:Group:Timepoint | | 1.663 | 0.831 | | 2.000 | | 439.540 | | 1.963 | | 0.142 | | 0.192 | |

| **Supplementary Table 1C. Inflammation** | | | | | | | |
| --- | --- | --- | --- | --- | --- | --- | --- |
| **IL6** | **Sum Sq** | **Mean Sq** | **NumDF** | **DenDF** | **F value** | **Pr(>F)** | **Significance without TCA** |
| Intervention | 0.143 | 0.143 | 1.000 | 240.470 | 1.120 | 0.291 | 0.340 |
| Group | 0.001 | 0.001 | 1.000 | 82.062 | 0.005 | 0.942 | 0.970 |
| Timepoint | 0.320 | 0.320 | 1.000 | 237.350 | 2.500 | 0.115 | 0.126 |
| Sex | 0.099 | 0.099 | 1.000 | 81.725 | 0.775 | 0.381 | 0.388 |
| Age | 0.020 | 0.020 | 1.000 | 82.404 | 0.154 | 0.696 | 0.780 |
| Testday | 0.141 | 0.141 | 1.000 | 242.535 | 1.107 | 0.294 | 0.362 |
| Stimulation Intensity | 0.138 | 0.138 | 1.000 | 273.977 | 1.083 | 0.299 | 0.365 |
| Intervention:Group | 0.389 | 0.389 | 1.000 | 239.317 | 3.044 | 0.082 | 0.105 |
| Interverntion:Timepoint | 0.007 | 0.007 | 1.000 | 237.347 | 0.057 | 0.812 | 0.652 |
| Group:Timepoint | 0.000 | 0.000 | 1.000 | 237.372 | 0.000 | 0.989 | 0.974 |
| Intervention:Group:Timepoint | 0.111 | 0.111 | 1.000 | 237.369 | 0.869 | 0.352 | 0.257 |
|  | | | | | | | |
| **TNF-alpha** | **Sum Sq** | **Mean Sq** | **NumDF** | **DenDF** | **F value** | **Pr(>F)** | **Significance without TCA** |
| Intervention | 0.186 | 0.186 | 1.000 | 231.112 | 0.574 | 0.450 | 0.515 |
| Group | 1.257 | 1.257 | 1.000 | 79.080 | 3.871 | 0.053 | 0.052 |
| Timepoint | 0.302 | 0.302 | 1.000 | 226.272 | 0.932 | 0.335 | 0.384 |
| Sex | 0.001 | 0.001 | 1.000 | 78.214 | 0.004 | 0.953 | 0.937 |
| Age | 0.690 | 0.690 | 1.000 | 79.291 | 2.125 | 0.149 | 0.140 |
| Testday | 0.034 | 0.034 | 1.000 | 233.186 | 0.104 | 0.747 | 0.825 |
| Stimulation Intensity | 0.184 | 0.184 | 1.000 | 281.579 | 0.568 | 0.452 | 0.525 |
| Intervention:Group | 0.003 | 0.003 | 1.000 | 230.015 | 0.010 | 0.922 | 0.880 |
| Interverntion:Timepoint | 0.029 | 0.029 | 1.000 | 226.250 | 0.089 | 0.765 | 0.824 |
| Group:Timepoint | 0.413 | 0.413 | 1.000 | 226.228 | 1.272 | 0.261 | 0.248 |
| Intervention:Group:Timepoint | 0.120 | 0.120 | 1.000 | 226.263 | 0.370 | 0.544 | 0.513 |

| **Supplementary Table 1D. Comparison of HRV baseline during the sham condition based on antidepressant class.** M=Mean, SD=Standard deviation, none=no Antidepressant, SSRI=Selective Serotonin Reuptake Inhibitor; SNRI=Serotonin and Norepinephrine Reuptake Inhibitors, TCA: Tricyclic Antidepressants, Other= any AD not falling into the categories of SSRI/SNRI/TCA. Significance Values: ***<0.001, **<0.01, *<0.05, #<0.1 | | | | | | | | |
| --- | --- | --- | --- | --- | --- | --- | --- | --- |
| **HRV variable** | **none** | **SSRI** | **SNRI** | **TCA** | **Other** | **missing** | **Kruskal Test p value** | **Dunn's test** |
| HF-HRV | M = 1213.02 (SD = 1493.23) | M = 671.72 (SD = 878.86) | M = 736.46 (SD = 1272.57) | M = 61.02 (SD = 50.15) | M = 4549.4 (SD = NA) | M = 1361.2 (SD = 1415.7) | **0.012*** | none-Other: p=0.255; none-SNRI: p=0.131; none-SSRI: p=0.077; **none-TCA:p=0.003****; SSRI-SNRI: p=0.891; SSRI-TCA: p=0.050; **SNRI-TCA:p=0.046***, |
| LF-HRV | M = 1417.26 (SD = 1172.76) | M = 1146.09 (SD = 1474.94) | M = 1014.14 (SD = 1049.92) | M = 659.98 (SD = 724.24) | M = 1345.04 (SD = NA) | M = 2123.69 (SD = 3191.65) | 0.457 | na |
| LFHFratio | M = 2.12 (SD = 1.49) | M = 6.08 (SD = 9.92) | M = 2.19 (SD = 1.54) | M = 11.32 (SD = 4.82) | M = 0.3 (SD = NA) | M = 2.13 (SD = 1.85) | 0.076 | na |
| Mean_HR | M = 68.21 (SD = 11.37) | M = 71.71 (SD = 11.17) | M = 70.36 (SD = 10.91) | M = 76.25 (SD = 7.88) | M = 54.54 (SD = NA) | M = 67.65 (SD = 9.47) | 0.331 | na |
| RMSSD_ms | M = 49.58 (SD = 32.77) | M = 38.14 (SD = 32.85) | M = 33.54 (SD = 21.54) | M = 15.6 (SD = 9.05) | M = 126.88 (SD = NA) | M = 53.24 (SD = 26.07) | **0.026*** | none-Other: p=0.276; none-SNRI: p=0.082; none-SSRI: p=0.148; **none-TCA:p=0.007****; SSRI-SNRI: p=0.709; SSRI-TCA: p=0.068; SNRI-TCA:p=0.120, |

| **Supplementary Table 2A. Demographic Characteristics of Participants Split by Cardiac Parasympathetic Activity (CPA) Group.** CTRL=Control group, MDD= Major Depressive Disorder group. Bold values indicate significant results.nr=not reported. Significance Values: ***<0.001, **<0.01, *<0.05, #<0.1 | | | | |
| --- | --- | --- | --- | --- |
|  | **N** | **CPA high**, N = 55*^1^* | **CPA low**, N = 54*^1^* | **p-value***^2^* |
| **Age** | 108 | 31.84 (11.90) | 37.72 (13.32) | **0.017 *** |
| **Sex** | 109 |  |  | >0.999 |
| female |  | 35 (64%) | 34 (63%) |  |
| male |  | 20 (36%) | 20 (37%) |  |
| **BMI** | 108 | 23.57 (3.14) | 24.27 (3.41) | 0.271 |
| **Group** | 109 |  |  | **<0.001 ***** |
| CTRL |  | 36 (65%) | 15 (28%) |  |
| MDD |  | 19 (35%) | 39 (72%) |  |
| **Smoker** | 109 |  |  | 0.273 |
| no |  | 42 (76%) | 44 (81%) |  |
| occasional |  | 7 (13%) | 2 (3.7%) |  |
| yes |  | 6 (11%) | 7 (13%) |  |
| unknown |  | 0 (0%) | 1 (1.9%) |  |
| **Contraception** | 108 |  |  | 0.496 |
| no |  | 20 (36%) | 21 (40%) |  |
| yes |  | 11 (20%) | 5 (9.4%) |  |
| postmenopausal |  | 3 (5.5%) | 4 (7.5%) |  |
| unknown |  | 1 (1.8%) | 3 (5.7%) |  |
| male |  | 20 (36%) | 20 (38%) |  |
| **Education** | 109 |  |  | 0.314 |
| 0- no degree |  | 0 (0%) | 2 (3.7%) |  |
| 1- primary school |  | 0 (0%) | 0 (0%) |  |
| 2- secondary school |  | 22 (40%) | 14 (26%) |  |
| 3- secondary education |  | 6 (11%) | 7 (13%) |  |
| 4- university degree |  | 27 (49%) | 29 (54%) |  |
| 5- doctoral degree |  | 0 (0%) | 1 (1.9%) |  |
| unknown |  | 0 (0%) | 1 (1.9%) |  |
| **unmedicated** | 108 |  |  | **0.039 *** |
| no |  | 14 (25%) | 26 (49%) |  |
| yes |  | 39 (71%) | 26 (49%) |  |
| nr |  | 2 (3.6%) | 1 (1.9%) |  |
| **antidepressants** | 109 |  |  | **0.001 **** |
| no |  | 45 (82%) | 26 (49%) |  |
| yes |  | 9 (16%) | 25 (47%) |  |
| nr |  | 2 (3.6%) | 2 (3.8%) |  |
| **Antidepressant type** |  |  |  | **0.1653** |
| SSRI/SNRI |  | 7(13%) | 21 (39%) |  |
| TCA |  | 0 (0%) | 3 (6%) |  |
| Other |  | 2 (4%) | 1(2%) |  |
| **CTQ Sum Score***^4^* | 107 | 36.76 (12.54) | 49.92 (19.26) | **<0.001 ***** |
| **PSS-10***^4^* | 108 | 15.15 (8.35) | 21.32 (8.70) | **<0.001 ***** |
| **MADRS***^4^* | 109 | 9.07 (12.48) | 19.44 (13.37) | **<0.001 ***** |
| **BDI-II***^4^* | 108 | 8.33 (11.43) | 19.33 (14.47) | **<0.001 ***** |
| **Baseline comparison (sham)** | | | | |
| **Positive Emotions (Composite Score)** | 103 | 269(73.3) | 215(76.7) | **<0.001***** |
| **Negative Emotions (Composite Score)** | 103 | 97.7(90.8) | 158(105.0) | **0.002**** |
| **Stress (%)** | 103 | 27.8(26.5) | 37.4(25.8) | 0.065 |
| **Median TNF-alpha in pg/mL (range)** | 86 | 0.37(0.07-1.86) | 0.41(0.07-5.45) | 0.924 |
| **Median IL-6 in pg/mL( range)** | 89 | 2.56(0.57-55.7) | 2.78(0.35-20.62) | 0.931 |
| **Mean HR** | 98 | 65.6(7.05) | 71.91(11.91) | **0.004**** |
| **RMSSD in ms** | 98 | 68.78(28.36) | 26.88(8.58) | **<0.001***** |
| **HF-HRV (FFT)** | 98 | 2010.4(1535.9) | 308.8(229.65) | **<0.001***** |
| **LF-HRV (FFT)** | 98 | 2582.1(3234.9) | 786.3(708.2) | **<0.001***** |
| *^1^* Mean (SD); n (%) | | | | |
| *^2^* Welch Two Sample t-test; Pearson’s Chi-squared test | | | | |
| *^1^* Mean (SD); n (%) | | | | |
| *^2^* Welch Two Sample t-test; Pearson’s Χ2 test | | | | |
| ^3^ P value derived from Chi-square test of MDD subgroup only | | | | |
| *^4^* Wilcoxon rank sum test | | | | |

| **Supplementary Table 2B. Demographic Characteristics of Control Participants split by CPA group. Bold values indicate significant results.nr=not reported.** Significance Values: ***<0.001, **<0.01, *<0.05, #<0.1 | | | | |
| --- | --- | --- | --- | --- |
|  | **N** | **high**, N = 36*^1^* | **low**, N = 15*^1^* | **p-value***^2^* |
| **Sample characteristics** | | | | |
| **Age** | 51 | 29.64 (10.13) | 31.67 (12.45) | 0.582 |
| **Sex** | 51 |  |  | 0.895 |
| female |  | 24 (67%) | 9 (60%) |  |
| male |  | 12 (33%) | 6 (40%) |  |
| **BMI** | 51 | 23.35 (2.49) | 24.11 (2.84) | 0.373 |
| **Smoker** | 51 |  |  | 0.139 |
| no |  | 28 (78%) | 15 (100%) |  |
| occasional |  | 4 (11%) | 0 (0%) |  |
| yes |  | 4 (11%) | 0 (0%) |  |
| unknown |  | 0 (0%) | 0 (0%) |  |
| **Contraception** | 51 |  |  | 0.796 |
| no |  | 13 (36%) | 6 (40%) |  |
| yes |  | 9 (25%) | 2 (13%) |  |
| postmenopausal |  | 1 (2.8%) | 0 (0%) |  |
| unknown |  | 1 (2.8%) | 1 (6.7%) |  |
| male |  | 12 (33%) | 6 (40%) |  |
| **Education** | 51 |  |  | 0.324 |
| 0- no degree |  | 0 (0%) | 0 (0%) |  |
| 1- primary school |  | 0 (0%) | 0 (0%) |  |
| 2- secondary school |  | 16 (44%) | 4 (27%) |  |
| 3- secondary education |  | 4 (11%) | 2 (13%) |  |
| 4- university degree |  | 16 (44%) | 8 (53%) |  |
| 5- doctoral degree |  | 0 (0%) | 1 (6.7%) |  |
| unknown |  | 0 (0%) | 0 (0%) |  |
| **Unmedicated** | 51 |  |  | 0.618 |
| no |  | 3 (8.3%) | 0 (0%) |  |
| yes |  | 33 (92%) | 15 (100%) |  |
| nr |  | 0 (0%) | 0 (0%) |  |
| **Antidepressants** | 51 |  |  |  |
| no |  | 36 (100%) | 15(100%) | na |
| yes |  | na | na |  |
| nr |  | na | na |  |
| **Questionnaires** | | | | |
| **BDI** |  | 0(8) | 0(9) | 0.47 |
| **MADRS** |  | 0(7) | 1(9) | 0.265 |
| **CTQ Sum Score** | 50 | 32.77 (10.09) | 37.53 (11.07) | **0.033 *** |
| **PSS 10** | 51 | 10.03 (4.44) | 11.20 (5.31) | 0.527 |
| **Baseline comparison (sham)** | | | | |
| **Positive Emotions (Composite Score)** | 50 | 305.47 (47.65) | 283.08 (63.28) | 0.271 |
| **Negative Emotions (Composite Score)** | 50 | 55.87 (54.73) | 72.67 (61.97) | 0.396 |
| **Stress (%)** | 50 | 16.88 (20.42) | 16.04 (17.44) | 0.983 |
| **Median TNF-alpha in pg/mL (range)** | 37 | 0.61 (1.79) | 1 (1.44) | 0.667 |
| **Median IL-6 in pg/mL( range)** | 40 | 2.27 (55.07) | 2.78 (9.26) | 0.510 |
| **Mean HR** | 49 | 65.48 (6.82) | 74.28 (10.64) | **0.004** |
| **RMSSD in ms** | 49 | 65.08 (24.46) | 29.41 (8.20) | **<0.001** |
| **HF-HRV (FFT)** | 49 | 1,868.13 (1,518.74) | 394.95 (237.60) | **<0.001** |
| **LF-HRV (FFT)** | 49 | 2,722.46 (3,805.84) | 982.43 (664.97) | **0.008** |
| *^1^* Mean (SD); n (%) | | | | |
| *^2^* Welch Two Sample t-test; Pearson’s Chi-squared test | | | | |

| **Supplementary Table 2C. Demographic Characteristics of Participants with MDD split by CPA group. Bold values indicate significant results.nr=not reported.** Significance Values: ***<0.001, **<0.01, *<0.05, #<0.1 | | | | |
| --- | --- | --- | --- | --- |
|  | **N** | **high**, N = 19*^1^* | **low**, N = 39*^1^* | **p-value***^2^* |
| **Sample characteristics** | | | | |
| **Age** | 57 | 36.00 (14.04) | 40.11 (13.04) | 0.294 |
| **Sex** | 58 |  |  | 0.866 |
| female |  | 11 (58%) | 25 (64%) |  |
| male |  | 8 (42%) | 14 (36%) |  |
| **BMI** | 57 | 24.00 (4.14) | 24.33 (3.64) | 0.767 |
| **Smoker** | 58 |  |  | 0.453 |
| no |  | 14 (74%) | 29 (74%) |  |
| occasional |  | 3 (16%) | 2 (5.1%) |  |
| yes |  | 2 (11%) | 7 (18%) |  |
| unknown |  | 0 (0%) | 1 (2.6%) |  |
| **Contraception** | 57 |  |  | 0.876 |
| no |  | 7 (37%) | 15 (39%) |  |
| yes |  | 2 (11%) | 3 (7.9%) |  |
| postmenopausal |  | 2 (11%) | 4 (11%) |  |
| unknown |  | 0 (0%) | 2 (5.3%) |  |
| male |  | 8 (42%) | 14 (37%) |  |
| **Education** | 58 |  |  | 0.787 |
| 0- no degree |  | 0 (0%) | 2 (5.1%) |  |
| 1- primary school |  | 0 (0%) | 0 (0%) |  |
| 2- secondary school |  | 6 (32%) | 10 (26%) |  |
| 3- secondary education |  | 2 (11%) | 5 (13%) |  |
| 4- university degree |  | 11 (58%) | 21 (54%) |  |
| 5- doctoral degree |  | 0 (0%) | 0 (0%) |  |
| unknown |  | 0 (0%) | 1 (2.6%) |  |
| **Unmedicated** | 57 |  |  | 0.418 |
| no |  | 11 (58%) | 26 (68%) |  |
| yes |  | 6 (32%) | 11 (29%) |  |
| nr |  | 2 (11%) | 1 (2.6%) |  |
| **Antidepressants** | 57 |  |  | 0.229 |
| no |  | 9 (47%) | 11 (29%) |  |
| yes |  | 8 (42%) | 25 (66%) |  |
| nr |  | 2 (11%) | 2 (5.3%) |  |
| **Questionnaires** | | | | |
| **BDI** | 57 | 22.78 (8.29) | 26.08 (11.02) | 0.192 |
| **MADRS** | 58 | 24.53 (8.76) | 26.21 (8.78) | 0.613 |
| **CTQ Sum Score** | 57 | 44.11 (13.52) | 54.82 (19.70) | **0.048 *** |
| **PSS 10** | 57 | 24.84 (4.39) | 25.32 (6.15) | 0.696 |
| **Baseline comparison (sham)** | | | | |
| **Positive Emotions (Composite Score)** | 54 | 187.88 (51.34) | 189.24 (64.46) | 0.894 |
| **Negative Emotions (Composite Score)** | 54 | 189.25 (87.44) | 188.50 (100.40) | 0.733 |
| **Stress (%)** | 54 | 51.56 (22.73) | 45.34 (23.68) | 0.251 |
| **Median TNF-alpha in pg/mL (range)** | | 0.28(0.7) | 0.35(5.39) | 0.685 |
| **Median IL-6 in pg/mL( range)** | 49 | 2.76 (9.37) | 2.8 (19.79) | 0.837 |
| **Mean HR** | 48 | 65.90 (7.80) | 70.99 (12.61) | 0.17 |
| **RMSSD in ms** | 48 | 77.20 (35.19) | 25.74 (8.76) | **<0.001** |
| **HF-HRV (FFT)** | 48 | 2,332.84 (1,578.31) | 272.68 (221.99) | **<0.001** |
| **LF-HRV (FFT)** | 48 | 2,263.97 (1,257.26) | 701.83 (729.83) | **<0.001** |
| *^1^* Mean (SD); n (%) | | | | |
| *^2^* Welch Two Sample t-test; Pearson’s Chi-squared test | | | | |

| **Supplementary Table 3A. Fixed Effects of lmer for Emotions in Type-III Anova Table.** Significance Values: ***<0.001, **<0.01, *<0.05, .<0.1. Formula: Emotion [transformed] ~ Intervention (sham/taVNS)*RMSSD(low/high)*Timepoint+ Group+ Sex+Age+ Testday+ Intensity+(1\| ID). Significance indicates the p value of all participants, the Significance without TCA indicates p values when those taking TCA were **omitted.** | | | | | | | |
| --- | --- | --- | --- | --- | --- | --- | --- |
| **Emotion: Stress** | **NumDF** | **DenDF** | **F** | **p value (Pr>F)** | **Significance** | **Significance without TCA** |  |
| Intervention | 1 | 507.07 | 0.26 | 0.612 |  | 0.182 |  |
| RMSSD(low/high) | 1 | 99.14 | 0.04 | 0.834 |  | 0.866 |  |
| Timepoint | 2 | 488.43 | 118.91 | **0.000** | ******* | **0.000** | ******* |
| Group | 1 | 98.76 | 38.23 | **0.000** | ******* | **0.000** | ******* |
| Sex | 1 | 97.56 | 0.37 | 0.544 |  | 0.777 |  |
| Age | 1 | 98.05 | 1.71 | 0.194 |  | 0.196 |  |
| Testday | 1 | 514.22 | 2.63 | 0.106 |  | 0.455 |  |
| Stimulation Intensity | 1 | 582.60 | 0.18 | 0.671 |  | 0.304 |  |
| Intervention:RMSSD(low/high) | 1 | 503.32 | 2.95 | 0.086 | **#** | **0.024** | ***** |
| Intervention:Timepoint | 2 | 488.43 | 0.93 | 0.394 |  | 0.366 |  |
| RMSSD(low/high):Timepoint | 2 | 488.43 | 2.38 | 0.094 | # | 0.186 |  |
| Intervention:RMSSD(low/high):Timepoint | 2 | 488.43 | 0.14 | 0.874 |  | 0.842 |  |
| **Emotion: Negative Composite Score** |  |  |  |  |  |  |  |
| Intervention | 1 | 501.94 | 1.70 | 0.192 |  | 0.576 |  |
| RMSSD(low/high) | 1 | 100.11 | 0.86 | 0.356 |  | 0.351 |  |
| Timepoint | 2 | 489.59 | 100.59 | **0.000** | ******* | **0.000** | ******* |
| Group | 1 | 99.89 | 58.71 | **0.000** | ******* | **0.000** | ******* |
| Sex | 1 | 99.10 | 3.38 | 0.069 | # | 0.113 |  |
| Age | 1 | 99.43 | 2.94 | 0.090 | # | 0.124 |  |
| Testday | 1 | 507.25 | 3.14 | 0.077 | # | 0.314 |  |
| Stimulation Intensity | 1 | 584.90 | 0.01 | 0.903 |  | 0.482 |  |
| Intervention:RMSSD(low/high) | 1 | 499.41 | 0.57 | 0.450 |  | 0.227 |  |
| Intervention:Timepoint | 2 | 489.59 | 1.48 | 0.228 |  | 0.232 |  |
| RMSSD(low/high):Timepoint | 2 | 489.59 | 2.32 | 0.099 | # | 0.191 |  |
| Intervention:RMSSD(low/high):Timepoint | 2 | 489.59 | 0.54 | 0.584 |  | 0.576 |  |
| **Emotion: Positive Composite Score** |  |  |  |  |  |  |  |
| Intervention | 1 | 501.51 | 1.08 | 0.300 |  | 0.808 |  |
| RMSSD(low/high) | 1 | 101.63 | 0.00 | 0.973 |  | 0.913 |  |
| Timepoint | 2 | 491.16 | 10.24 | **0.000** | ******* | **0.000** | ******* |
| Group | 1 | 101.44 | 98.68 | **0.000** | ******* | **0.000** | ******* |
| Sex | 1 | 506.09 | 10.68 | 0.001 | ** | 0.644 |  |
| Age | 1 | 101.05 | 0.96 | 0.329 |  | 0.404 |  |
| Testday | 1 | 100.77 | 0.52 | 0.473 |  | 0.016 | * |
| Stimulation Intensity | 1 | 576.94 | 0.80 | 0.372 |  | 0.800 |  |
| Intervention:RMSSD(low/high) | 1 | 499.38 | 2.01 | 0.157 |  | 0.340 |  |
| Intervention:Timepoint | 2 | 491.16 | 0.14 | 0.874 |  | 0.855 |  |
| RMSSD(low/high):Timepoint | 2 | 491.16 | 2.32 | 0.099 | # | 0.060 | # |
| Intervention:RMSSD(low/high):Timepoint | 2 | 491.16 | 0.04 | 0.960 |  | 0.929 |  |

| **Supplementary Table 3B. Fixed Effects of lmer for Emotions in Type-III Anova Table with RMSSD as continuous moderator.** Significance Values: ***<0.001, **<0.01, *<0.05, #<0.1. Formula: Emotion [transformed] ~ Intervention(sham/taVNS)*RMSSD(continuous)*Timepoint+ Group+ Sex+ Age+ Testday+ Intensity+ (1\| ID). Significance indicates the p value of all participants, the Significance without TCA indicates p values when those taking TCA were omitted. | | | | | | | |
| --- | --- | --- | --- | --- | --- | --- | --- |
| **Stressed** | **NumDF** | **DenDF** | **F** | **p value (Pr>F)** | **Significance** | **Significance without TCA** |  |
| Intervention | 1 | 496.400 | 2.773 | 0.097 | # | **0.006** | ****** |
| RMSSD(continuous) | 1 | 98.860 | 1.537 | 0.218 |  | 0.264 |  |
| Timepoint | 2 | 488.510 | 23.408 | **< 0.001** | ******* | **0.000** | ******* |
| Group | 1 | 98.910 | 38.110 | **< 0.001** | ******* | **0.000** | ******* |
| Sex | 1 | 97.580 | 0.321 | 0.573 |  | 0.789 |  |
| Age | 1 | 98.010 | 1.681 | 0.198 |  | 0.202 |  |
| Testday | 1 | 512.490 | 1.975 | 0.161 |  | 0.686 |  |
| Stimulation Intensity | 1 | 582.720 | 0.414 | 0.520 |  | 0.182 |  |
| Intervention:RMSSD(continuous) | 1 | 499.530 | 2.381 | 0.123 |  | **0.020** | ***** |
| Intervention:Timepoint | 2 | 488.510 | 0.451 | 0.637 |  | 0.590 |  |
| RMSSD(continuous):Timepoint | 2 | 488.440 | 0.838 | 0.433 |  | 0.698 |  |
| Intervention:RMSSD(continuous):Timepoint | 2 | 488.440 | 0.036 | 0.965 |  | 0.947 |  |
| **Negative** | **NumDF** | **DenDF** | **F** | **p value (Pr>F)** | **Significance** |  |  |
| Intervention | 1 | 494.480 | 0.724 | 0.395 |  | 0.962 |  |
| RMSSD(continuous) | 1 | 99.820 | 1.530 | 0.219 |  | 0.219 |  |
| Timepoint | 2 | 489.540 | 15.979 | **0.000** | ******* | **0.000** | ******* |
| Group | 1 | 99.900 | 62.939 | **0.000** | ******* | **0.000** | ******* |
| Sex | 1 | 99.020 | 3.185 | 0.077 | # | 0.122 |  |
| Age | 1 | 99.300 | 2.541 | 0.114 |  | 0.159 |  |
| Testday | 1 | 505.810 | 3.010 | 0.083 | # | 0.362 |  |
| Stimulation Intensity | 1 | 584.450 | 0.050 | 0.824 |  | 0.398 |  |
| Intervention:RMSSD(continuous) | 1 | 496.490 | 0.051 | 0.822 |  | 0.725 |  |
| Intervention:Timepoint | 2 | 489.540 | 2.367 | 0.095 | # | 0.090 | # |
| RMSSD(continuous):Timepoint | 2 | 489.500 | 2.497 | 0.083 | # | 0.177 |  |
| Intervention:RMSSD(continuous):Timepoint | 2 | 489.500 | 1.046 | 0.352 |  | 0.319 |  |
| **Positive** | **NumDF** | **DenDF** | **F** | **p value (Pr>F)** | **Significance** |  |  |
| Intervention (sham/taVNS) | 1 | 495.250 | 2.831 | 0.093 | # | 0.544 |  |
| RMSSD(continuous) | 1 | 101.430 | 0.173 | 0.679 |  | 0.686 |  |
| Timepoint | 2 | 491.170 | 0.417 | 0.659 |  | 0.790 |  |
| Group | 1 | 101.510 | 106.331 | **< 0.001** | ******* | **<0.001** | ******* |
| Sex | 1 | 504.920 | 11.978 | **0.001** | ******* | 0.640 |  |
| Age | 1 | 101.000 | 0.962 | 0.329 |  | 0.402 |  |
| Testday | 1 | 100.750 | 0.541 | 0.464 |  | **0.012** | ***** |
| Stimulation Intensity | 1 | 576.410 | 1.184 | 0.277 |  | 0.695 |  |
| Intervention:RMSSD(continuous) | 1 | 496.920 | 1.544 | 0.215 |  | 0.600 |  |
| Intervention:Timepoint | 2 | 491.170 | 0.055 | 0.946 |  | 0.961 |  |
| RMSSD(continuous):Timepoint | 2 | 491.140 | 1.604 | 0.202 |  | 0.135 |  |
| Intervention:RMSSD(continuous):Timepoint | 2 | 491.140 | 0.035 | 0.966 |  | 0.996 |  |

| **Supplementary Table 4A. Fixed Effects of lmer for ECG data in Type-III Anova Table.** All ECG measures were log transformed for analyses. Significance Values: ***<0.001, **<0.01, *<0.05, .<0.1. Formula: log(ECG measure) ~ Intervention(sham/taVNS)*RMSSD(low/high)*Timepoint+ Group+ Sex+ Age+ Testday+ Intensity+ (1\| ID). Significance indicates the p value of all participants, the Significance without TCA indicates p values when those taking TCA were omitted. | | | | | | | |
| --- | --- | --- | --- | --- | --- | --- | --- |
|  | **NumDF** | **DenDF** | **F** | **p value (Pr>F)** | **Significance** | **Significance without TCA** |  |
| Intervention | 1 | 455.640 | 0.228 | 0.633 |  | 0.549 |  |
| RMSSD(low/high) | 1 | 100.690 | 6.210 | **0.014** | ***** | **0.023** | ***** |
| Timepoint | 2 | 444.030 | 58.656 | **0.000** | ******* | **0.000** | ******* |
| Group | 1 | 100.250 | 0.039 | 0.844 |  | 0.951 |  |
| Sex | 1 | 99.980 | 12.357 | **0.001** | ******* | **0.000** | ******* |
| Age | 1 | 100.640 | 11.283 | **0.001** | ****** | **0.001** | ******* |
| Testday | 1 | 459.850 | 0.091 | 0.764 |  | 0.653 |  |
| Stimulation Intensity | 1 | 513.310 | 2.402 | 0.122 |  | 0.091 | . |
| Intervention:RMSSD(low/high) | 1 | 453.810 | 2.409 | 0.121 |  | 0.108 |  |
| Intervention:Timepoint | 2 | 443.910 | 0.460 | 0.632 |  | 0.666 |  |
| RMSSD(low/high):Timepoint | 2 | 444.020 | 4.041 | **0.018** | ***** | **0.042** | ***** |
| Intervention:RMSSD(low/high):Timepoint | 2 | 443.920 | 0.175 | 0.840 |  | 0.808 |  |
| **RMSSD** |  |  |  |  |  |  |  |
| Intervention | 1 | 460.680 | 2.784 | 0.096 | # | 0.222 |  |
| RMSSD(low/high) | 1 | 99.800 | 57.264 | 0.000 | ******* | **0.000** | ******* |
| Timepoint | 2 | 443.400 | 27.502 | 0.000 | ******* | **0.000** | ******* |
| Group | 1 | 99.100 | 0.339 | 0.562 |  | 0.706 |  |
| Sex | 1 | 98.720 | 1.980 | 0.162 |  | 0.413 |  |
| Age | 1 | 99.700 | 0.113 | 0.737 |  | 0.973 |  |
| Testday | 1 | 466.430 | 0.163 | 0.686 |  | 0.926 |  |
| Stimulation Intensity | 1 | 534.900 | 0.811 | 0.368 |  | 0.601 |  |
| Intervention:RMSSD(low/high) | 1 | 458.000 | 13.485 | 0.000 | ******* | **0.000** | ******* |
| Intervention:Timepoint | 2 | 443.210 | 1.806 | 0.165 |  | 0.190 |  |
| RMSSD(low/high):Timepoint | 2 | 443.380 | 6.373 | 0.002 | ****** | **0.003** | ****** |
| Intervention:RMSSD(low/high):Timepoint | 2 | 443.210 | 0.930 | 0.395 |  | 0.434 |  |
| **HF-HRV** |  |  |  |  |  |  |  |
| Intervention | 1 | 465.460 | 1.111 | 0.292 |  | 0.460 |  |
| RMSSD(low/high) | 1 | 99.770 | 48.815 | 0.000 | ******* | **0.000** | ******* |
| Timepoint | 2 | 443.610 | 15.089 | 0.000 | ******* | **0.000** | ******* |
| Group | 1 | 98.820 | 0.832 | 0.364 |  | 0.472 |  |
| Sex | 1 | 98.350 | 7.423 | 0.008 | ****** | **0.030** | ***** |
| Age | 1 | 99.600 | 2.729 | 0.102 |  | 0.169 |  |
| Testday | 1 | 472.180 | 1.784 | 0.182 |  | 0.347 |  |
| Stimulation Intensity | 1 | 542.750 | 1.371 | 0.242 |  | 0.369 |  |
| Intervention:RMSSD(low/high) | 1 | 462.150 | 9.327 | 0.002 | ****** | **0.001** | ******* |
| Intervention:Timepoint | 2 | 443.360 | 0.316 | 0.729 |  | 0.786 |  |
| RMSSD(low/high):Timepoint | 2 | 443.590 | 6.208 | 0.002 | ****** | **0.004** | ****** |
| Intervention:RMSSD(low/high):Timepoint | 2 | 443.370 | 1.416 | 0.244 |  | 0.315 |  |
| **LF-HRV** |  |  |  |  |  |  |  |
| Intervention | 1 | 470.940 | 1.852 | 0.174 |  | 0.274 |  |
| RMSSD(low/high) | 1 | 98.140 | 19.360 | **0.000** | ******* | **0.000** | ******* |
| Timepoint | 2 | 442.440 | 23.449 | **0.000** | ******* | **0.000** | ******* |
| Group | 1 | 96.810 | 0.699 | 0.405 |  | 0.461 |  |
| Sex | 1 | 96.230 | 0.395 | 0.531 |  | 0.188 |  |
| Age | 1 | 97.870 | 10.344 | **0.002** | ****** | **0.002** | ****** |
| Testday | 1 | 478.680 | 0.746 | 0.388 |  | 0.586 |  |
| Stimulation Intensity | 1 | 534.250 | 1.758 | 0.185 |  | 0.287 |  |
| Intervention:RMSSD(low/high) | 1 | 466.730 | 4.996 | **0.026** | ***** | **0.016** | ***** |
| Intervention:Timepoint | 2 | 442.090 | 3.227 | **0.041** | ***** | **0.045** | ***** |
| RMSSD(low/high):Timepoint | 2 | 442.410 | 1.693 | 0.185 |  | 0.161 |  |
| Intervention:RMSSD(low/high):Timepoint | 2 | 442.090 | 0.273 | 0.761 |  | 0.794 |  |
| **LF/HF ratio** |  |  |  |  |  |  |  |
| Intervention | 1.00 | 472.860 | 0.118 | 0.731 |  | 0.720 |  |
| RMSSD(low/high) | 1.00 | 95.700 | 12.549 | **0.001** | ******* | **0.001** | ****** |
| Timepoint | 2.00 | 440.240 | 1.911 | 0.149 |  | 0.234 |  |
| Group | 1.00 | 94.150 | 0.045 | 0.833 |  | 0.958 |  |
| Sex | 1.00 | 93.510 | 15.365 | **0.000** | ******* | **0.000** | ******* |
| Age | 1.00 | 95.350 | 2.433 | 0.122 |  | 0.065 | . |
| Testday | 1.00 | 481.060 | 0.172 | 0.679 |  | 0.717 |  |
| Stimulation Intensity | 1.00 | 518.340 | 0.013 | 0.910 |  | 0.889 |  |
| Intervention:RMSSD(low/high) | 1.00 | 468.110 | 0.630 | 0.428 |  | 0.416 |  |
| Intervention:Timepoint | 2.00 | 439.820 | 1.581 | 0.207 |  | 0.183 |  |
| RMSSD(low/high):Timepoint | 2.00 | 440.190 | 2.073 | 0.127 |  | 0.226 |  |
| Intervention:RMSSD(low/high):Timepoint | 2.00 | 439.820 | 1.823 | 0.163 |  | 0.225 |  |

| **Supplementary Table 4B. Follow-Up contrasts for significant interactions of HRV (post-hoc).** All ECG measures were log transformed for analyses. Post-hoc tests on model without those taking tricyclic antidepressants. Significance Values: ***<0.001, **<0.01, *<0.05, .<0.1. Formula: log(ECG measure) ~ Intervention(sham/taVNS)*RMSSD(low/high)*Timepoint+ Group+ Sex+ Age+ Testday+ Intensity+ (1\| ID). BL=baseline, PS= Stress, REC=Recovery | | | | | | | |
| --- | --- | --- | --- | --- | --- | --- | --- |
| **Mean HR** | **RMSSD Group** | **Contrast** | **estimate** | **SE** | **df** | **t ratio** | **p value** |
| RMSSD(low/high):Timepoint | High RMSSD | BL-PS | -0.084 | 0.009 | 433.000 | -9.117 | **<0.001** |
|  |  | BL-REC | -0.023 | 0.009 | 433.000 | -2.525 | **0.032** |
|  |  | PS-REC | 0.061 | 0.009 | 432.000 | 6.622 | **<0.001** |
|  | Low RMSSD | BL-PS | -0.052 | 0.009 | 433.000 | -5.759 | **<0.001** |
|  |  | BL-REC | -0.015 | 0.009 | 433.000 | -1.574 | 0.258 |
|  |  | PS-REC | 0.038 | 0.009 | 433.000 | 4.125 | **0.000** |
| **RMSSD** |  |  |  |  |  |  |  |
| RMSSD(low/high):Timepoint | High RMSSD | BL-PS | 0.272 | 0.038 | 433.000 | 7.082 | **<0.001** |
|  |  | BL-REC | 0.061 | 0.038 | 434.000 | 1.590 | 0.251 |
|  |  | PS-REC | -0.211 | 0.038 | 433.000 | -5.515 | **<0.001** |
|  | Low RMSSD | BL-PS | 0.085 | 0.038 | 433.000 | 2.233 | **0.067** |
|  |  | BL-REC | -0.030 | 0.038 | 434.000 | -0.774 | 0.719 |
|  |  | PS-REC | -0.114 | 0.038 | 433.000 | -2.983 | **0.009** |
| Intervention:RMSSD(low/high) | High RMSSD | sham-tVNS | 0.122 | 0.034 | 457.000 | 3.580 | **0.000** |
|  | Low RMSSD | sham-tVNS | -0.064 | 0.033 | 444.000 | -1.964 | **0.050** |
| **HF-HRV** |  |  |  |  |  |  |  |
| RMSSD(low/high):Timepoint | High RMSSD | BL-PS | 0.552 | 0.089 | 434.000 | 6.197 | **<0.001** |
|  |  | BL-REC | 0.256 | 0.089 | 434.000 | 2.883 | **0.012** |
|  |  | PS-REC | -0.296 | 0.089 | 433.000 | -3.334 | **0.003** |
|  | Low RMSSD | BL-PS | 0.133 | 0.088 | 433.000 | 1.515 | 0.285 |
|  |  | BL-REC | 0.030 | 0.089 | 434.000 | 0.338 | 0.939 |
|  |  | PS-REC | -0.103 | 0.089 | 433.000 | -1.162 | 0.477 |
| Intervention:RMSSD(low/high) | High RMSSD | sham-tVNS | 0.219 | 0.079 | 463.000 | 2.786 | **0.006** |
|  | Low RMSSD | sham-tVNS | -0.138 | 0.075 | 447.000 | -1.835 | **0.067** |
| **LF-HRV** |  |  |  |  |  |  |  |
| Intervention:Timepoint | sham | BL-PS | 0.283 | 0.097 | 434.000 | 2.906 | **0.011** |
|  |  | BL-REC | 0.121 | 0.097 | 433.000 | 1.249 | 0.425 |
|  |  | PS-REC | -0.162 | 0.097 | 433.000 | -1.663 | 0.221 |
|  | tVNS | BL-PS | 0.566 | 0.095 | 434.000 | 5.939 | **<0.001** |
|  |  | BL-REC | 0.098 | 0.096 | 436.000 | 1.014 | 0.568 |
|  |  | PS-REC | -0.468 | 0.096 | 434.000 | -4.864 | **<0.001** |
| Intervention:RMSSD(low/high) | High RMSSD | sham-tVNS | 0.207 | 0.085 | 470.000 | 2.423 | **0.016** |
|  | **Low RMSSD** | sham-tVNS | -0.076 | 0.082 | 451.000 | -0.927 | 0.354 |
| **LF/HF ratio** |  |  |  |  |  |  |  |
| no significant interaction |  |  |  |  |  |  |  |
|  |  |  |  |  |  |  |  |

| **Supplementary Table 4C. Fixed Effects of lmer for ECG data in Type-III Anova Table where RMSSD(continuous) was used as moderator.** RMSSD (continuous) indicates RMSSD (baseline, sham) as continuous variable. All ECG measures were log transformed for analyses. Significance Values: ***<0.001, **<0.01, *<0.05, #<0.1. Formula: log(ECG measure) ~ Intervention(sham/taVNS)*RMSSD(continuous)*Timepoint+ Group+ Sex+ Age+ Testday+ Intensity+ (1\| ID). Significance indicates the p value of all participants, the Significance without TCA indicates p values when those taking TCA were omitted. | | | | | | | |
| --- | --- | --- | --- | --- | --- | --- | --- |
| **HR** | **NumDF** | **DenDF** | **F** | **p value (Pr>F)** | **Significance** | **Significance without TCA, continuous CPA** |  |
| Intervention | 1.000 | 450.850 | 2.938 | 0.087 | # | 0.058 | # |
| RMSSD(continuous) | 1.000 | 100.430 | 8.049 | **0.006** | ****** | **0.014** | ***** |
| Timepoint | 2.000 | 443.990 | 7.296 | **0.001** | ******* | **0.000** | ******* |
| Group | 1.000 | 100.280 | 0.130 | 0.720 |  | 0.807 |  |
| Sex | 1.000 | 99.890 | 13.393 | **< 0.001** | ******* | **0.000** | ******* |
| Age | 1.000 | 100.410 | 9.086 | **0.003** | ****** | **0.002** | ****** |
| Testday | 1.000 | 459.780 | 0.232 | 0.630 |  | 0.498 |  |
| Stimulation Intensity | 1.000 | 513.280 | 3.278 | 0.071 | # | **0.049** | ***** |
| Intervention:RMSSD(continuous) | 1.000 | 450.820 | 2.630 | 0.106 |  | 0.075 | # |
| Intervention:Timepoint | 2.000 | 443.950 | 0.423 | 0.656 |  | 0.616 |  |
| RMSSD(continuous):Timepoint | 2.000 | 443.950 | 1.940 | 0.145 |  | 0.284 |  |
| Intervention:RMSSD(continuous):Timepoint | 2.000 | 443.840 | 0.423 | 0.656 |  | 0.608 |  |
| **RMSSD** | **NumDF** | **DenDF** | **F** | **p value (Pr>F)** | **Significance** | **Significance without TCA, continuous CPA** |  |
| Intervention | 1.000 | 458.340 | 17.995 | **< 0.001** | ******* | **0.000** | ******* |
| RMSSD(continuous) | 1.000 | 101.300 | ###### | **< 0.001** | ******* | **< 0.001** | ******* |
| Timepoint | 2.000 | 445.460 | 2.568 | **0.078** | **#** | **0.098** | **#** |
| Group | 1.000 | 100.840 | 1.231 | 0.270 |  | 0.330 |  |
| Sex | 1.000 | 100.140 | 1.411 | 0.238 |  | 0.562 |  |
| Age | 1.000 | 101.120 | 3.311 | 0.072 | # | 0.126 |  |
| Testday | 1.000 | 472.870 | 0.031 | 0.860 |  | 0.355 |  |
| Stimulation Intensity | 1.000 | 542.400 | 0.068 | 0.794 |  | 0.733 |  |
| Intervention:RMSSD(continuous) | 1.000 | 458.380 | 32.512 | **< 0.001** | ******* | **0.000** | ******* |
| Intervention:Timepoint | 2.000 | 445.370 | 1.190 | 0.305 |  | 0.362 |  |
| RMSSD(continuous):Timepoint | 2.000 | 445.410 | 16.659 | **< 0.001** | ******* | **0.000** | ******* |
| Intervention:RMSSD(continuous):Timepoint | 2.000 | 445.190 | 0.122 | 0.886 |  | 0.905 |  |
| **HF-HRV** | **NumDF** | **DenDF** | **F** | **p value (Pr>F)** | **Significance** | **Significance without TCA, continuous CPA** |  |
| Intervention | 1.000 | 460.890 | 13.465 | **< 0.001** | ******* | **< 0.001** | ******* |
| RMSSD(continuous) | 1.000 | 101.450 | 87.881 | **< 0.001** | ******* | **< 0.001** | ******* |
| Timepoint | 2.000 | 445.850 | 1.442 | 0.238 |  | 0.237 |  |
| Group | 1.000 | 100.830 | 2.120 | 0.148 |  | 0.186 |  |
| Sex | 1.000 | 100.040 | 7.090 | **0.009** | ****** | **0.034** | ***** |
| Age | 1.000 | 101.180 | 9.669 | **0.002** | ****** | **0.004** | ****** |
| Testday | 1.000 | 476.950 | 0.740 | 0.390 |  | 0.789 |  |
| Stimulation Intensity | 1.000 | 542.100 | 0.383 | 0.537 |  | 0.868 |  |
| Intervention:RMSSD(continuous) | 1.000 | 460.980 | 21.855 | **< 0.001** | ******* | **< 0.001** | ******* |
| Intervention:Timepoint | 2.000 | 445.750 | 1.188 | 0.306 |  | 0.412 |  |
| RMSSD(continuous):Timepoint | 2.000 | 445.810 | 13.782 | **< 0.001** | ******* | **< 0.001** | ******* |
| Intervention:RMSSD(continuous):Timepoint | 2.000 | 445.550 | 0.752 | 0.472 |  | 0.550 |  |
| **LF-HRV** | **NumDF** | **DenDF** | **F** | **p value (Pr>F)** | **Significance** | **Significance without TCA, continuous CPA** |  |
| Intervention | 1.000 | 460.890 | 0.383 | 0.536 |  | 0.355 |  |
| RMSSD(continuous) | 1.000 | 97.610 | 27.276 | **0.000** | ******* | **< 0.001** | ******* |
| Timepoint | 2.000 | 442.410 | 3.094 | **0.046** | ***** | 0.130 | ns |
| Group | 1.000 | 96.760 | 1.254 | 0.266 |  | 0.285 |  |
| Sex | 1.000 | 95.890 | 0.738 | 0.392 |  | 0.136 |  |
| Age | 1.000 | 97.200 | 15.977 | 0.000 | ******* | **< 0.001** | ******* |
| Testday | 1.000 | 479.220 | 0.545 | 0.461 |  | 0.704 |  |
| Stimulation Intensity | 1.000 | 532.130 | 0.727 | 0.394 |  | 0.588 |  |
| Intervention:RMSSD(continuous) | 1.000 | 461.070 | 1.852 | 0.174 |  | 0.122 | ns |
| Intervention:Timepoint | 2.000 | 442.270 | 0.176 | 0.839 |  | 0.895 |  |
| RMSSD(continuous):Timepoint | 2.000 | 442.380 | 3.961 | 0.020 | * | 0.010 | * |
| Intervention:RMSSD(continuous):Timepoint | 2.000 | 442.050 | 0.649 | 0.523 |  | 0.508 |  |
| **LF/HF-ratio** | **NumDF** | **DenDF** | **F** | **p value (Pr>F)** | **Significance** | **Significance without TCA, continuous CPA** |  |
| Intervention | 1.000 | 463.160 | 7.668 | **0.006** | ****** | **0.004** | ****** |
| RMSSD(continuous) | 1.000 | 96.250 | 20.608 | **0.000** | ******* | **< 0.001** | ******* |
| Timepoint | 2.000 | 441.450 | 6.734 | **0.001** | ****** | **0.005** | ****** |
| Group | 1.000 | 95.130 | 0.164 | 0.687 |  | 0.797 |  |
| Sex | 1.000 | 94.160 | 15.426 | **0.000** | ******* | **< 0.001** | ******* |
| Age | 1.000 | 95.660 | 1.071 | 0.303 |  | 0.188 |  |
| Testday | 1.000 | 483.040 | 0.006 | 0.938 |  | 0.921 |  |
| Stimulation Intensity | 1.000 | 511.440 | 0.109 | 0.741 |  | 0.678 |  |
| Intervention:RMSSD(continuous) | 1.000 | 463.450 | 8.480 | **0.004** | ****** | **0.002** | ****** |
| Intervention:Timepoint | 2.000 | 441.280 | 0.772 | 0.463 |  | 0.572 |  |
| RMSSD(continuous):Timepoint | 2.000 | 441.450 | 4.523 | **0.011** | ***** | **0.024** | ***** |
| Intervention:RMSSD(continuous):Timepoint | 2.000 | 441.040 | 2.435 | 0.089 | # | 0.108 |  |

| **Supplementary Table 4D. Fixed Effects of lmer for ECG data in Type-III Anova Table including the Age*Intervention (sham/taVNS) interaction.** All ECG measures were log transformed for analyses. Significance Values: ***<0.001, **<0.01, *<0.05, .<0.1. Formula: log(ECG measure) ~ Intervention(sham/taVNS)*RMSSD(low/high)*Timepoint+ Intervention(sham/taVNS)* Age+Group+ Sex+ Age+ Testday+ Intensity+ (1\| ID). Analyses were perfomred without patients on TCA. | | | | | | |
| --- | --- | --- | --- | --- | --- | --- |
| **RMSSD as a dicotomous moderator** | | | | | | **RMSSD as continous predictor** |
| **HR** | **Num DF** | **DenDF** | **F** | **p value (Pr>F)** | **Significance** | **Significance with RMSSD as continuous predictor** |
| Intervention(sham/taVNS) | 1 | 441.45 | 4.803 | **0.029** | ***** | ***** |
| RMSSD(low/high) | 1 | 97.55 | 5.347 | **0.023** | ***** | ***** |
| Timepoint | 2 | 431.95 | 59.214 | **<0.001** | ******* | ******* |
| Age | 1 | 97.49 | 12.980 | **<0.001** | ******* | ****** |
| Group | 1 | 97.2 | 0.008 | 0.928 |  |  |
| Sex | 1 | 96.89 | 15.546 | **<0.001** | ******* | ******* |
| Testday | 1 | 448.66 | 0.159 | 0.691 |  |  |
| Stimulation Intensity | 1 | 502.05 | 3.368 | 0.067 | # | ***** |
| Intervention(sham/taVNS):RMSSD(low/high) | 1 | 442.98 | 4.583 | **0.033** | ***** | ***** |
| Intervention(sham/taVNS):Timepoint | 2 | 431.82 | 0.415 | 0.661 |  |  |
| RMSSD(low/high):Timepoint | 2 | 431.93 | 3.130 | **0.045** | ***** |  |
| Intervention(sham/taVNS):Age | 1 | 442.23 | 4.472 | 0.035 | * | # |
| Intervention(sham/taVNS):RMSSD(low/high):Timepoint | 2 | 431.82 | 0.235 | 0.791 |  |  |
| **RMSSD** | **NumDF** | **DenDF** | **F** | **p value (Pr>F)** | **Significance** | **Significance with RMSSD as continuous predictor** |
| Intervention(sham/taVNS) | 1 | 446.68 | 23.970 | **<0.001** | ******* | ******* |
| RMSSD(low/high) | 1 | 96.12 | 64.166 | **<0.001** | ******* | ******* |
| Timepoint | 2 | 430.89 | 27.796 | **<0.001** | ******* |  |
| Age | 1 | 95.97 | 0.020 | 0.888 |  |  |
| Group | 1 | 95.47 | 0.241 | 0.625 |  |  |
| Sex | 1 | 94.99 | 0.561 | 0.456 |  |  |
| Testday | 1 | 457.54 | 0.002 | 0.967 |  |  |
| Stimulation Intensity | 1 | 525.94 | 0.027 | 0.869 |  |  |
| Intervention(sham/taVNS):RMSSD(low/high) | 1 | 449.2 | 30.698 | **<0.001** | ******* | ******* |
| Intervention(sham/taVNS):Timepoint | 2 | 430.67 | 1.795 | 0.167 |  |  |
| RMSSD(low/high):Timepoint | 2 | 430.85 | 6.113 | **0.002** | ****** | ******* |
| Intervention(sham/taVNS):Age | 1 | 447.99 | 32.482 | **<0.001** | ******* | ******* |
| Intervention(sham/taVNS):RMSSD(low/high):Timepoint | 2 | 430.66 | 0.953 | 0.386 |  |  |
| **HF-HRV** | **NumDF** | **DenDF** | **F** | **p value (Pr>F)** | **Significance** | **Significance with RMSSD as continuous predictor** |
| Intervention(sham/taVNS) | 1 | 451.32 | 11.887 | **0.001** | ******* | ******* |
| RMSSD(low/high) | 1 | 95.86 | 53.807 | **<0.001** | ******* | ******* |
| Timepoint | 2 | 430.98 | 15.155 | **<0.001** | ******* |  |
| Age | 1 | 95.61 | 1.766 | 0.187 |  | ****** |
| Group | 1 | 94.94 | 0.670 | 0.415 |  |  |
| Sex | 1 | 94.33 | 4.734 | **0.032** | ***** | ***** |
| Testday | 1 | 464.21 | 1.100 | 0.295 |  |  |
| Stimulation Intensity | 1 | 525.78 | 0.434 | 0.510 |  |  |
| Intervention(sham/taVNS):RMSSD(low/high) | 1 | 454.54 | 18.835 | **0.000** | ******* | ******* |
| Intervention(sham/taVNS):Timepoint | 2 | 430.68 | 0.253 | 0.777 |  |  |
| RMSSD(low/high):Timepoint | 2 | 430.94 | 5.567 | **0.004** | ****** | ******* |
| Intervention(sham/taVNS):Age | 1 | 453.01 | 15.701 | **<0.001** | ******* | ******* |
| Intervention(sham/taVNS):RMSSD(low/high):Timepoint | 2 | 430.68 | 1.256 | 0.286 |  |  |
| **LF-HRV** | **NumDF** | **DenDF** | **F** | **p value (Pr>F)** | **Significance** | **Significance with RMSSD as continuous predictor** |
| Intervention(sham/taVNS) | 1 | 454.76 | 1.549 | 0.214 |  |  |
| RMSSD(low/high) | 1 | 94.03 | 20.087 | **<0.001** | ******* | ******* |
| Timepoint | 2 | 429.56 | 20.823 | **<0.001** | ******* |  |
| Age | 1 | 93.64 | 10.393 | **0.002** | ****** | ******* |
| Group | 1 | 92.81 | 0.610 | 0.437 |  |  |
| Sex | 1 | 92.08 | 1.854 | 0.177 |  |  |
| Testday | 1 | 469.39 | 0.344 | 0.558 |  |  |
| Stimulation Intensity | 1 | 507.53 | 0.912 | 0.340 |  |  |
| Intervention(sham/taVNS):RMSSD(low/high) | 1 | 458.7 | 7.910 | **0.005** | ****** | . |
| Intervention(sham/taVNS):Timepoint | 2 | 429.16 | 3.152 | **0.044** | ***** |  |
| RMSSD(low/high):Timepoint | 2 | 429.5 | 1.804 | 0.166 |  | ***** |
| Intervention (sham/taVNS):Age | 1 | 456.87 | 3.025 | 0.083 | # |  |
| Intervention:RMSSD(low/high):Timepoint | 2 | 429.16 | 0.208 | 0.812 |  |  |
| **LF/HF ratio** | **NumDF** | **DenDF** | **F** | **p value (Pr>F)** | **Significance** | **Significance with RMSSD as continuous predictor** |
| Intervention(sham/taVNS) | 1 | 455.7 | 4.172 | **0.042** | ***** | ****** |
| RMSSD(low/high) | 1 | 92.87 | 11.385 | **0.001** | ****** | ******* |
| Timepoint | 2 | 428.56 | 1.492 | 0.226 |  | ****** |
| Age | 1 | 92.43 | 3.768 | 0.055 | # |  |
| Group | 1 | 91.53 | 0.011 | 0.915 |  |  |
| Sex | 1 | 90.77 | 14.432 | **<0.001** | ******* | ******* |
| Testday | 1 | 470.93 | 0.172 | 0.679 |  |  |
| Stimulation Intensity | 1 | 495.83 | 0.072 | 0.789 |  |  |
| Intervention (sham/taVNS):RMSSD(low/high) | 1 | 459.93 | 1.805 | 0.180 |  | ****** |
| Intervention (sham/taVNS):Timepoint | 2 | 428.12 | 1.704 | 0.183 |  |  |
| RMSSD(low/high):Timepoint | 2 | 428.49 | 1.452 | 0.235 |  | ***** |
| Intervention (sham/taVNS):Age | 1 | 457.97 | 4.191 | 0.041 | ***** | ***** |
| Intervention (sham/taVNS):RMSSD(low/high):Timepoint | 2 | 428.12 | 1.560 | 0.211 |  |  |

| **Supplementary Table 4E.** Estimated Marginal means by age. Results are averaged over the levels of: RMSSD(low/high), Timepoint, Group, Sex, Testday. Degrees-of-freedom method: kenward-roger. Results are given on the log scale. Significance Values: ***<0.001, **<0.01, *<0.05, #<0.1 | | | | | |
| --- | --- | --- | --- | --- | --- |
| **Index** | **Age** | **Contrast (sham − tVNS)** | **Interpretation** | **p-value** | **Significance** |
| **HR** | 20 | 0.017 | ns | 0.050 | # |
|  | 30 | 0.007 | ns | 0.238 |  |
|  | 40 | −0.0025 | ns | 0.695 |  |
|  | 50 | −0.0121 | ns | 0.196 |  |
|  | 60 | −0.0217 | ns | 0.101 |  |
| **RMSSD** | 20 | −0.115 | tVNS > sham | **<0.001** | ******* |
|  | 30 | −0.010 | ns | 0.667 |  |
|  | 40 | 0.094 | sham > tVNS | **<.001** | ******* |
|  | 50 | 0.198 | sham > tVNS | **<.001** | ******* |
|  | 60 | 0.303 | sham > tVNS | **<.001** | ******* |
| **HF-HRV** | 20 | −0.195 | tVNS > sham | **0.016** | ***** |
|  | 30 | −0.024 | ns | 0.673 |  |
|  | 40 | 0.147 | sham > tVNS | **0.016** | ***** |
|  | 50 | 0.317 | sham > tVNS | **<.001** | ******* |
|  | 60 | 0.488 | sham > tVNS | **<.001** | ******* |
| **LF-HRV** | ns | | | |  |
| **LF/HF** | 20 | 0.155 | ns | 0.081 |  |
|  | 30 | 0.058 | ns | 0.351 |  |
|  | 40 | −0.038 | ns | 0.564 |  |
|  | 50 | −0.135 | ns | 0.165 |  |
|  | 60 | −0.232 | ns | 0.093 |  |

| **Supplementary Table 5A. Fixed Effects of lmer for Inflammation data in Type-III Anova Table.** All Cytokines were transformed with a Tukey ladder of power transformations for analyses. Significance Values: ***<0.001, **<0.01, *<0.05, #<0.1 Formula: Cytokine [transformed] ~ Intervention(sham/taVNS)*RMSSD(low/high)*Timepoint+ Group+ Sex+ Age+ Testday+ Intensity+ (1\| ID). Significance without TCA indicates p values when those taking TCA were omitted. | | | | | | |
| --- | --- | --- | --- | --- | --- | --- |
| **TNF-α[log]** | **NumDF** | **DenDF** | **F** | **Significance** | **Significance no TCA** |  |
| Intervention | 1 | 233.538 | 1.344 | 0.248 | 0.324 |  |
| RMSSD(low/high) | 1 | 78.412 | 0.428 | 0.515 | 0.552 |  |
| Timepoint | 1 | 226.329 | 0.834 | 0.362 | 0.403 |  |
| Group | 1 | 78.252 | 4.119 | **0.046** | **0.047** | ***** |
| Sex | 1 | 77.278 | 0.001 | 0.974 | 0.948 |  |
| Age | 1 | 78.431 | 2.328 | 0.131 | 0.128 |  |
| Testday | 1 | 234.166 | 0.381 | 0.538 | 0.648 |  |
| Stimulation Intensity | 1 | 281.034 | 1.417 | 0.235 | 0.300 |  |
| Intervention:RMSSD(low/high) | 1 | 231.801 | 4.363 | **0.038** | **0.038** | ***** |
| Intervention:Timepoint | 1 | 226.334 | 0.042 | 0.839 | 0.876 |  |
| RMSSD(low/high):Timepoint | 1 | 226.302 | 0.311 | 0.578 | 0.557 |  |
| Intervention:RMSSD(low/high):Timepoint | 1 | 226.302 | 0.071 | 0.790 | 0.826 |  |
| **IL-6[log]** |  |  |  |  |  |  |
| Intervention | 1 | 241.925 | 1.668 | 0.198 | 0.282 |  |
| RMSSD(low/high) | 1 | 81.504 | 0.097 | 0.757 | 0.787 |  |
| Timepoint | 1 | 237.435 | 2.081 | 0.151 | 0.160 |  |
| Group | 1 | 81.055 | 0.000 | 1.000 | 0.982 |  |
| Sex | 1 | 80.725 | 0.751 | 0.389 | 0.396 |  |
| Age | 1 | 81.561 | 0.109 | 0.742 | 0.816 |  |
| Testday | 1 | 242.454 | 0.919 | 0.339 | 0.471 |  |
| Stimulation Intensity | 1 | 274.112 | 0.897 | 0.345 | 0.453 |  |
| Intervention:RMSSD(low/high) | 1 | 240.440 | 0.525 | 0.469 | 0.422 |  |
| Intervention:Timepoint | 1 | 237.413 | 0.121 | 0.728 | 0.606 |  |
| RMSSD(low/high):Timepoint | 1 | 237.424 | 0.134 | 0.714 | 0.728 |  |
| Intervention:RMSSD(low/high):Timepoint | 1 | 237.428 | 0.019 | 0.891 | 0.964 |  |

| **Supplementary Table 5B. Fixed Effects of lmer for Inflammation data in Type-III Anova Table where RMSSD(continuous) was used as moderator.** RMSSD (continuous) indicates RMSSD (baseline, sham) as continuous variable. All Cytokines were transformed with a Tukey ladder of power transformations for analyses. Significance Values: ***<0.001, **<0.01, *<0.05, #<0.1. Formula: Cytokine [transformed] ~ Intervention*RMSSD(continuous)*Timepoint+ Group+ Sex+ Age+ Testday+ Intensity+ (1\| ID). Significance indicates the p value of all participants, the Significance without TCA indicates p values when those taking TCA were omitted. | | | | | | | |
| --- | --- | --- | --- | --- | --- | --- | --- |
| **TNF-α[transformed]** | **NumDF** | **DenDF** | **F** | **p value (Pr>F)** | **Significance** | **Significance without TCA, continuous CPA** |  |
| Intervention | 1 | 228.878 | 0.387 | 0.534 |  | 0.459 |  |
| RMSSD(continuous) | 1 | 80.239 | 2.579 | 0.112 |  | 0.128 |  |
| Timepoint | 1 | 226.411 | 0.033 | 0.856 |  | 0.948 |  |
| Group | 1 | 78.484 | 5.180 | 0.026 | * | **0.028** | ***** |
| Sex | 1 | 77.037 | 0.024 | 0.877 |  | 0.926 |  |
| Age | 1 | 78.472 | 2.241 | 0.138 |  | 0.142 |  |
| Testday | 1 | 232.669 | 0.091 | 0.763 |  | 0.893 |  |
| Stimulation Intensity | 1 | 281.002 | 0.711 | 0.400 |  | 0.492 |  |
| Intervention:RMSSD(continuous) | 1 | 230.686 | 1.438 | 0.232 |  | 0.209 |  |
| Intervention:Timepoint | 1 | 226.540 | 0.531 | 0.467 |  | 0.506 |  |
| RMSSD(continuous):Timepoint | 1 | 226.673 | 0.108 | 0.743 |  | 0.678 |  |
| Intervention:RMSSD(continuous):Timepoint | 1 | 226.770 | 0.527 | 0.469 |  | 0.490 |  |
| **IL6 [transformed]** | **NumDF** | **DenDF** | **F** | **p value (Pr>F)** |  |  |  |
| Intervention | 1 | 238.666 | 0.076 | 0.783 |  | 0.524 |  |
| RMSSD(continuous) | 1 | 83.959 | 0.154 | 0.696 |  | 0.636 |  |
| Timepoint | 1 | 238.148 | 3.415 | 0.066 | # | 0.073 | # |
| Group | 1 | 81.287 | 0.028 | 0.868 |  | 0.890 |  |
| Sex | 1 | 80.607 | 0.674 | 0.414 |  | 0.414 |  |
| Age | 1 | 81.517 | 0.138 | 0.711 |  | 0.796 |  |
| Testday | 1 | 241.790 | 0.686 | 0.409 |  | 0.609 |  |
| Stimulation Intensity | 1 | 272.564 | 0.725 | 0.395 |  | 0.556 |  |
| Intervention:RMSSD(continuous) | 1 | 239.667 | 1.127 | 0.290 |  | 0.186 |  |
| Intervention:Timepoint | 1 | 238.153 | 0.217 | 0.642 |  | 0.412 |  |
| RMSSD(continuous):Timepoint | 1 | 239.100 | 1.548 | 0.215 |  | 0.221 |  |
| Intervention:RMSSD(continuous):Timepoint | 1 | 239.107 | 0.105 | 0.746 |  | 0.532 |  |
