# Supplementary material for: The heart knows best: baseline heart rate variability as guide to transcutaneous auricular vagus nerve stimulation in depression
Source: Transl Psychiatry. 2025 Dec 6;15:521. doi: 10.1038/s41398-025-03780-y (PMC12689627; doi:10.1038/s41398-025-03780-y)
Supplement: Supplementary file 2 — Supplementary Information [file 41398_2025_3780_MOESM2_ESM.docx]

**Supplementary Information**

**HRV-Analysis including antidepressant medication (yes/no)**

When including antidepressant medication (yes/no) in the models, antidepressant use was not a significant predictor for any of the HRV variables except for LF-HRV (p=0.032). All Interactions stayed significant/remained insignificant compared to the models reported in **Supplementary table 4A**. In these models, MADRS was used instead of group as none oft he participants in the healthy contorl group took antidepressants.

| Heart Rate | Sum Sq | Mean Sq | NumDF | DenDF | F value | Pr(>F) | Sig |
| --- | --- | --- | --- | --- | --- | --- | --- |
| Intervention | 0.001 | 0.001 | 1 | 455.370 | 0.201 | 0.654 |  |
| RMSSD(low/high) | 0.016 | 0.016 | 1 | 98.350 | 4.347 | 0.040 | * |
| Timepoint | 0.438 | 0.219 | 2 | 443.830 | 58.612 | <0.001 | *** |
| MADRS | <0.001 | <0.001 | 1 | 97.440 | 0.004 | 0.948 |  |
| Sex | 0.043 | 0.043 | 1 | 97.920 | 11.489 | 0.001 | ** |
| Age | 0.044 | 0.044 | 1 | 98.530 | 11.764 | 0.001 | *** |
| Testday | <0.001 | <0.001 | 1 | 459.710 | 0.068 | 0.794 |  |
| Stimulation Intensity | 0.008 | 0.008 | 1 | 513.300 | 2.223 | 0.137 |  |
| antidepressants | **0.011** | **0.006** | **2** | **98.520** | **1.534** | **0.221** |  |
| Intervention:RMSSD(low/high) | 0.009 | 0.009 | 1 | 453.470 | 2.474 | 0.116 |  |
| Intervention:Timepoint | 0.003 | 0.002 | 2 | 443.710 | 0.456 | 0.634 |  |
| RMSSD(low/high):Timepoint | 0.030 | 0.015 | 2 | 443.810 | 4.041 | 0.018 | * |
| Intervention:RMSSD(low/high):Timepoint | 0.001 | 0.001 | 2 | 443.700 | 0.173 | 0.842 |  |

| RMSSD | Sum Sq | Mean Sq | NumDF | DenDF | F value | Pr(>F) | Sig |
| --- | --- | --- | --- | --- | --- | --- | --- |
| Intervention | 0.193 | 0.193 | 1 | 460.730 | 2.950 | 0.087 | . |
| RMSSD(low/high) | 3.401 | 3.401 | 1 | 97.410 | 51.917 | <0.001 | *** |
| Timepoint | 3.599 | 1.800 | 2 | 443.230 | 27.473 | <0.001 | *** |
| MADRS | 0.027 | 0.027 | 1 | 96.020 | 0.404 | 0.527 |  |
| Sex | 0.111 | 0.111 | 1 | 96.740 | 1.697 | 0.196 |  |
| Age | 0.004 | 0.004 | 1 | 97.660 | 0.064 | 0.801 |  |
| Testday | 0.015 | 0.015 | 1 | 466.730 | 0.235 | 0.628 |  |
| Stimulation Intensity | 0.064 | 0.064 | 1 | 535.000 | 0.976 | 0.324 |  |
| antidepressants | **0.355** | **0.178** | **2** | **97.650** | **2.711** | **0.071** | **.** |
| Intervention:RMSSD(low/high) | 0.892 | 0.892 | 1 | 457.910 | 13.623 | <0.001 | *** |
| Intervention:Timepoint | 0.237 | 0.118 | 2 | 443.040 | 1.806 | 0.166 |  |
| RMSSD(low/high):Timepoint | 0.835 | 0.418 | 2 | 443.200 | 6.376 | 0.002 | ** |
| Intervention:RMSSD(low/high):Timepoint | 0.121 | 0.060 | 2 | 443.030 | 0.922 | 0.399 |  |
| HF-HRV | **Sum Sq** | **Mean Sq** | **NumDF** | **DenDF** | **F value** | **Pr(>F)** | **Sig** |
| Intervention | 0.428 | 0.428 | 1 | 465.460 | 1.215 | 0.271 |  |
| RMSSD(low/high) | 15.827 | 15.827 | 1 | 97.550 | 44.901 | <0.001 | *** |
| Timepoint | 10.632 | 5.316 | 2 | 443.670 | 15.081 | <0.001 | *** |
| MADRS | 0.085 | 0.085 | 1 | 95.790 | 0.241 | 0.625 |  |
| Sex | 2.452 | 2.452 | 1 | 96.680 | 6.957 | 0.010 | ** |
| Age | 0.946 | 0.946 | 1 | 97.830 | 2.685 | 0.105 |  |
| Testday | 0.691 | 0.691 | 1 | 472.380 | 1.959 | 0.162 |  |
| Stimulation Intensity | 0.543 | 0.543 | 1 | 540.970 | 1.541 | 0.215 |  |
| antidepressants | **1.537** | **0.769** | **2** | **97.840** | **2.181** | **0.118** |  |
| Intervention:RMSSD(low/high) | 3.312 | 3.312 | 1 | 462.020 | 9.396 | 0.002 | ** |
| Intervention:Timepoint | 0.223 | 0.112 | 2 | 443.430 | 0.317 | 0.729 |  |
| RMSSD(low/high):Timepoint | 4.382 | 2.191 | 2 | 443.640 | 6.216 | 0.002 | ** |
| Intervention:RMSSD(low/high):Timepoint | 0.987 | 0.494 | 2 | 443.420 | 1.400 | 0.248 |  |

| LF-HRV | Sum Sq | Mean Sq | NumDF | DenDF | F value | Pr(>F) | Sig |
| --- | --- | --- | --- | --- | --- | --- | --- |
| Intervention | 0.868 | 0.868 | 1 | 471.830 | 2.079 | 0.150 |  |
| RMSSD(low/high) | 6.560 | 6.560 | 1 | 96.090 | 15.712 | <0.001 | *** |
| Timepoint | 19.549 | 9.774 | 2 | 442.840 | 23.410 | <0.001 | *** |
| MADRS | 0.030 | 0.030 | 1 | 93.730 | 0.071 | 0.791 |  |
| Sex | 0.146 | 0.146 | 1 | 94.890 | 0.350 | 0.556 |  |
| Age | 4.165 | 4.165 | 1 | 96.410 | 9.974 | 0.002 | ** |
| Testday | 0.382 | 0.382 | 1 | 479.830 | 0.915 | 0.339 |  |
| Stimulation Intensity | 0.906 | 0.906 | 1 | 527.400 | 2.170 | 0.141 |  |
| antidepressants | **2.985** | **1.493** | **2** | **96.440** | **3.575** | **0.032** | ***** |
| Intervention:RMSSD(low/high) | 2.176 | 2.176 | 1 | 467.400 | 5.211 | 0.023 | * |
| Intervention:Timepoint | 2.688 | 1.344 | 2 | 442.480 | 3.219 | 0.041 | * |
| RMSSD(low/high):Timepoint | 1.434 | 0.717 | 2 | 442.790 | 1.717 | 0.181 |  |
| Intervention:RMSSD(low/high):Timepoint | 0.240 | 0.120 | 2 | 442.480 | 0.287 | 0.751 |  |

| LF/HF ratio | Sum Sq | Mean Sq | NumDF | DenDF | F value | Pr(>F) | Sig |
| --- | --- | --- | --- | --- | --- | --- | --- |
| Intervention | 0.054 | 0.054 | 1 | 472.360 | 0.129 | 0.720 |  |
| RMSSD(low/high) | 5.725 | 5.725 | 1 | 93.770 | 13.574 | <0.001 | *** |
| Timepoint | 1.611 | 0.806 | 2 | 440.670 | 1.910 | 0.149 |  |
| MADRS | 0.025 | 0.025 | 1 | 91.270 | 0.060 | 0.807 |  |
| Sex | 5.774 | 5.774 | 1 | 92.480 | 13.691 | <0.001 | *** |
| Age | 0.872 | 0.872 | 1 | 94.080 | 2.066 | 0.154 |  |
| Testday | 0.066 | 0.066 | 1 | 480.710 | 0.157 | 0.692 |  |
| Stimulation Intensity | 0.010 | 0.010 | 1 | 516.690 | 0.024 | 0.876 |  |
| antidepressants | **0.323** | **0.162** | **2** | **94.130** | **0.383** | **0.683** |  |
| Intervention:RMSSD(low/high) | 0.246 | 0.246 | 1 | 467.560 | 0.583 | 0.446 |  |
| Intervention:Timepoint | 1.328 | 0.664 | 2 | 440.270 | 1.574 | 0.208 |  |
| RMSSD(low/high):Timepoint | 1.747 | 0.873 | 2 | 440.620 | 2.071 | 0.127 |  |
| Intervention:RMSSD(low/high):Timepoint | 1.538 | 0.769 | 2 | 440.270 | 1.823 | 0.163 |  |
