# Supplementary material for: The heart knows best: baseline heart rate variability as guide to transcutaneous auricular vagus nerve stimulation in depression
Source: Transl Psychiatry. 2025 Dec 6;15:521. doi: 10.1038/s41398-025-03780-y (PMC12689627; doi:10.1038/s41398-025-03780-y)

Supplementary Figure 1. Pre-calculated power calculation for effect sizes. At 80% power,an effec size of 0.152 can be detected. Graph made with GPower software.

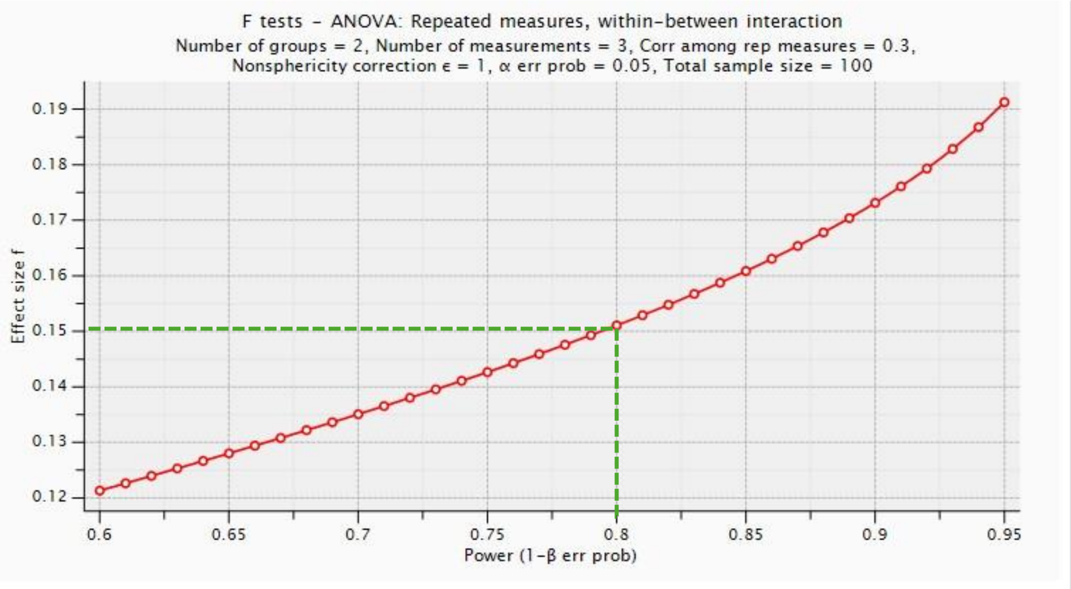

Supplement: Supplementary file 3 — Supplementary Figure 1 [file 41398_2025_3780_MOESM3_ESM.pdf]
